# Use of elemental profiles determined by energy-dispersive X-ray fluorescence and multivariate analyses to detect adulteration in Ceylon cinnamon

**DOI:** 10.1007/s00216-023-04817-1

**Published:** 2023-08-17

**Authors:** Michele Ghidotti, Sergej Papoci, Danilo Pietretti, Tereza Ždiniaková, María Beatriz de la Calle Guntiñas

**Affiliations:** European Commission, Joint Research Centre (JRC), Geel, Belgium

**Keywords:** *Cinnamomum verum*, Cassia cinnamon, EDXRF, GC–MS, Multivariate analysis, Food fraud

## Abstract

The price of *Cinnamomum verum* (Ceylon cinnamon) is around twice as high as that of the other cinnamon varieties commonly grouped under the name cassia cinnamon, making the former spice an attractive target for fraudsters. This work demonstrates that elemental profiles obtained by energy-dispersive X-ray fluorescence in combination with multivariate analyses can be used as a screening method to detect Ceylon cinnamon adulteration. Thirty-six elements were analysed in 52 commercially available cinnamon samples, 29 Ceylon, 8 cassia, and 15 for which no indication about variety was provided. Fifty-eight percent of the samples were either adulterated or did not meet international quality criteria. Four of the ground cinnamon samples labelled as Ceylon cinnamon were found to be pure cassia or a mixture with a high cassia content, and 26 samples were suspected of other types of adulteration including replacement of bark with other parts of the cinnamon tree. Headspace gas chromatography–mass spectrometry and ash determination by thermogravimetric analysis confirmed the conclusions reached by elemental analysis. Only one sample labelled as Ceylon cinnamon and that according to its volatile composition was cassia cinnamon was not flagged as suspicious by elemental analysis.

**Graphical Abstract:**

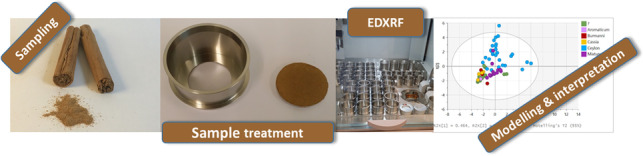

**Supplementary Information:**

The online version contains supplementary material available at 10.1007/s00216-023-04817-1.

## Introduction

According to the European Parliament Resolution of 14 January 2014 on the food crisis, fraud in the food chain, and the control thereof [1], spices are one of the food commodities most affected by fraudulent practices.

The main consumers of spices are Asians and Europeans. While 81% of herbs and spices worldwide are produced in Asia, only 2% are produced in Europe that imports three times the amounts of spices it produces [2].

Some spices are not only used as culinary ingredients but are also consumed for their health benefits. This is for instance the case of cinnamon that could have antioxidant, antitumor, cardiovascular, and cholesterol-lowing effects, and that could help to combat diabetes mellitus [3].

The genus *Cinnamomum*, which belongs to the Laureaceae family, has around 250 species. The most delicate and appreciated cinnamon in the international market is the inner bark of the tree *Cinnamomum verum* (also known as *Cinnamomum zeylanicum* Blume or Ceylon cinnamon), which originates from Sri Lanka and the Indian Malabar Coast, although it also grows in Madagascar and The Seychelles. Cassia is a spice similar to cinnamon, of lesser quality, grouping *Cinnamomum aromaticum* (China), *Cinnamomum loureiroi* (Vietnam), *Cinnamomum burmanii* (Indonesia, Sumatra, and Java), and *Cinnamomum tamala* (India and Myanmar) [4, 5]. Substitution of *C*. *verum* with cassia is a known type of adulteration because the price of the more delicate spice *C*. *verum* is around twice as high as that of cassia. From the point of view of food safety, the consumption of high amounts of cassia should be avoided because it contains coumarin, a substance that cannot be used as food additive because of its toxicity, and that is absent or present in low amounts in Ceylon cinnamon. Accordingly, analytical methods are needed to detect the total or partial substitution of *C*. *verum* with cassia. Other types of *C*. *verum* adulteration consist in the substitution of the bark powder with sugar, ground walnut shells, and powdered beechnut shells aromatised with cinnamaldehyde [4].

The volatile composition of *C*. *verum* has been thoroughly studied and reported in the literature [6–8]. The main volatile compound is cinnamaldehyde (around 80% of the bark oil). Other volatiles present in *C*. *verum* are eugenol, eugenol acetate, cinnamyl acetate, cinnamyl alcohol, methyl eugenol, benzaldehyde, benzyl benzoate, linalool, caryophillene, safrole, pinene, phylandrene, cymene, cineol, and others [4, 6]. The relative volatile composition of the oil extracted from the different parts of the plant is different, with camphor being the most abundant compound in root bark oil (around 60%) and the leaves oil being richer in eugenol (70 to 95%) than the bark oil (5 to 10%).

The analysis of volatile compounds allows to differentiate *C*. *verum* and cassia, because the former contains eugenol and benzyl benzoate which are absent in the latter, and because coumarin and δ-cadinene, present in cassia, are either absent or present in small amounts in *C*. *verum* [7]. Therefore, gas chromatography-mass spectrometry (GC–MS) can be used to detect substitution of *C*. *verum* with cassia [9], as well as extension of ground bark with other parts of the plant. Similar studies have been carried out using liquid chromatography-mass spectrometry (LC–MS) [10]. Frequently, GC–MS requires time-consuming sample treatment involving extraction of the compounds of interest with organic solvents. Extractions can be avoided with special devices such as headspace (HS) coupled to the gas chromatograph. Nevertheless, HS-GC–MS instruments are expensive and not widely available in all control laboratories. From an analytical point of view, due to the high number of volatile compounds present in cinnamon, it is expensive and tedious to construct calibration curves that would allow the quantification of all those compounds. Semi-quantitative analyses, using existing databases for interpretation of mass spectra, can be done but it requires a certain level of expertise and does not support comparison of results obtained by different laboratories, jeopardising the use of central databases for authentication purposes.

Another group of methods for classification of *C. verum* and cassia are based on untargeted infrared (IR) spectroscopic analysis in combination with multivariate data analysis for modelling purposes [11, 12]. Lack of standardisation is an important drawback linked to the use of IR. Other approaches used to differentiate *C. verum* and cassia are nuclear magnetic resonance (NMR) [13] based on metabolomics and chemometrics and DNA analysis [14].

The analytical system for the determination of major and trace elements is strongly developed and robust. Most food control laboratories are equipped for the analyses of major and trace elements; standardisation and quality control are supported by the availability of certified reference materials (CRMs); and comparability of results is warranted by the regular participation of laboratories in proficiency tests. Several works have been published on the determination of elements in spices, including cinnamon, using different techniques such as wavelength-dispersive X-ray fluorescence (WD-XRF) [15], total reflection X-ray fluorescence (TXRF) [16], inductively coupled plasma-mass spectrometry (ICP-MS) [17], a combination of ICP-MS and ICP-atomic emission spectrometry (ICP-AES) [18], and flame atomic absorption spectrometry (FAAS) [19]. Nevertheless, to the best of our knowledge, no work has been published so far on the detection of fraudulent substitution of *C. verum* with cassia, based on their elemental profiles.

A big advantage of energy-dispersive X-ray fluorescence (EDXRF) is that samples do not need to be digested before analyses, what eliminates the need to handle strong acids and reduces the amount of hazardous waste produced. Hand-held devices and portable EDXRF instrument are commercially available and can be used for in-situ analyses. Major and trace element profiles determined by EDXRF have been successfully used to authenticate different types of food, such as rice [20], coconut sugar [21], and honey [22], based on their botanical variety and with the support of multivariate analysis.

The aim of this work was to test whether the elemental profiles of *C. verum* and cassia are different enough to detect the substitution of the former by the latter, as well as other types of adulteration such as replacement of bark with other parts of the cinnamon. The elemental profiles of 52 commercially available cinnamon samples obtained by EDXRF were used in combination with multivariate analysis to build authentication models. Only commercially available cinnamon samples were included in the study; if some of the *C. verum* and cassia samples would be adulterated, the information obtained by EDXRF about the elemental profiles of the two spices would not be conclusive and it could jeopardise the full study if the rate of adulterated samples would be high. To avoid this risk, the conclusions based on elemental analysis on the authenticity of the *C. verum* samples were crosschecked with, and confirmed by, the results obtained with volatile compounds determined by HS-GC–MS and thermogravimetric analysis (TGA) for determining the ash content.

## Materials and methods

### Cinnamon samples

Thirty-seven commercially available powder cinnamon samples were purchased in Belgian, Italian, Maltese, Spanish, and Slovakian supermarkets of different chains and small retailers, to avoid the risk of all the samples belonging to the same batch/original producer. Four samples were sold as bulk and the remaining were pre-packaged and branded as Austrian, Belgian, Italian, Israeli, French, Maltese, Dutch, Spanish, Slovakian, and USA registered trade marks. Fourteen samples were purchased on-line, all of them commercialised by registered trade marks.

According to the information provided on the labels, 29 samples were *C. verum* referred to as Ceylon cinnamon in this work, 3 *C. burmanii*, 1 *C. aromaticum*, and 4 cassia cinnamon; for the remaining 15, no information about botanical variety was provided. Regarding the origin of the cinnamon, 14 were from Sri Lanka, 3 from Madagascar, 2 from Vietnam, 1 from India, 1 from Indonesia, and 1 was a mixture of cinnamon from Sri Lanka and Indonesia. For the remaining 30 samples, the only information given on the label about geographical origin was that the product was issue of non-EU agriculture. Information about the cinnamon samples included in this study is summarised in Table 1. Two Ceylon cinnamon samples, 45 and 51, were of the same brand.

**Table 1:** Information on the analysed samples. Total ash content calculated by TGA and conclusions reached by EDXRF, TGA, and HS-GC–MS analysis

Sample id	Comment	Label variety	Bark/ground	Country of origin	Continent	Purchased in	Ash (%) TGA	STD (%)	Conclusion HS-GC–MS	Conclusion EDXRF	Conclusion TGA
1		NA	Ground	NA	Asia	Belgium	3.9	0.19			
2		NA	Ground	NA	NON EU	Belgium	4.6	0.12			
3		NA	Ground	NA	NON EU	France	4.0	0.01			
4		NA	Ground	NA	NON EU	Belgium	4.5	0.003			
5		NA	Ground	NA	NON EU	France	5.1	0.03	S	S	S
6		NA	Ground	NA	NON EU	Netherlands	4.6	0.26			
7		NA	Ground	NA	NON EU	Belgium	4.2	0.07			
8		NA	Ground	NA	NON EU	Sweden	4.6	0.06			
9		NA	Ground	NA	NON EU	USA	4.0	0.06			
10		NA	Ground	NA	NON EU	Israel	3.2	0.07	S	S	
11	Organic	Ceylon	Ground	Madagascar	Africa	Austria	4.0	0.27	S	S	
12	Organic	Ceylon	Ground	Sri Lanka	Asia	Netherlands	4.2	1.0			
13	Organic	NA	Ground	NA	NON EU	Belgium	5.0	0.04			
14	Organic	Ceylon	Ground	Madagascar	Africa	France	4.5	0.01	S	S	
15	Organic	Burmanni	Ground	NA	NON EU	Belgium	4.5	0.03			
16	Organic	Aromaticum	Ground	NA	NON EU	Netherlands	4.4	0.01			S
17		NA	Ground	Ceylan and Indonesia	Asia	Spain	4.3	0.15	S	S	
18		Ceylon	Ground	NA	NON EU	Spain	5.1	0.60	S	S	
19	Organic	Ceylon	Ground	NA	NON EU	Spain	3.2	0.51	S	S	
20	Organic	Ceylon	Ground	NA	NON EU	Spain	4.9	0.19	S	S	
21	Bulk	Ceylon	Ground	NA	NON EU	Spain	6.3	0.10	S	S	S
22	Bulk	Ceylon	Ground	NA	NON EU	Spain	5.6	0.12	S	S	S
23		Cassia	Ground	NA	NON EU	Spain	4.4	0.13			
24	Organic	Cassia	Ground	Vietnam	Asia	Austria	4.2	0.01			
25		Ceylon	Ground	NA	NON EU	Slovakia	5.7	0.09		S	S
26		NA	Ground	Vietnam	Asia	Slovakia	4.4	0.74	S	S	
27		Burmanni	Ground		NON EU	Italy	4.2	0.02			
28	Organic	Burmanni	Ground		NON EU	Italy	4.6	0.27			
29	Bulk	Ceylon	Ground	NA	NON EU	Belgium	6.4	0.62		S	S
30	Bulk	Cassia	Ground	NA	NON EU	Belgium	4.1	0.10			
31		NA	Ground	Sri Lanka	Asia	Malta	3.9	0.26			
32		Ceylon	Bark	Sri Lanka	Asia	Netherlands	4.7	0.15			
33		Ceylon	Bark	Sri Lanka	Asia	Belgium	5.6	0.66		S	S
34		Ceylon	Bark	Sri Lanka	Asia	Netherlands	4.8	0.43			
35	Organic	Ceylon	Bark	Sri Lanka	Asia	France	6.2	0.28		S	S
36	Organic	Ceylon	Bark	Sri Lanka	Asia	Austria	6.5	0.06		S	S
37	Organic	Ceylon	Bark	NA	NON EU	Belgium	5.3	0.14		S	S
38	Bulk	Ceylon	Bark	NA	NA	Spain	5.2	0.09		S	S
39		Ceylon	Bark	Sri Lanka	Asia	UK	6.5	0.25	S	S	S
40	Organic	Ceylon	Ground	NA	NON EU	Germany	3.9	0.34	S	S	
41		Ceylon	Ground	Sri Lanka	Asia	Spain	4.3	0.08			
42		Ceylon	Bark	Sri Lanka	Asia	Spain	5.5	0.07		S	S
43	Organic	Ceylon	Ground	NA	NON EU	Spain	5.8	0.02	S	S	S
44		Ceylon	Ground	Madagascar	Africa	France	3.5	0.42	S	S	
45		Ceylon	Ground	Sri Lanka	Asia	Bulgaria	4.9	0.03	S	S	
46	Organic	Ceylon	Ground	India	Asia	Spain	3.9	0.01	S		
47	Organic	Ceylon	Bark	NA	Non EU	Belgium	4.1	0.01	S	S	
48	Organic	Ceylon	Bark	Sri Lanka	Asia	Germany	4.8	0.10			
49	Organic	Ceylon	Ground	NA	Non EU	Germany	3.8	0.18	S	S	
50		Ceylon	Ground	Sri Lanka	Asia	UK	4.7	0.09	S	S	
51		Ceylon	Ground	Sri Lanka	Asia	Bulgaria	4.9	0.01	S	S	
52		Cassia	Bark	Indonesia	Asia	Belgium	4.9	0.18			

*NA* not available, *S* suspicious

Forty samples were ground (according to the labels 19 *C. verum*, 2 *C. burmanii*, and three were just labelled as cassia; no information on botanical variety was provided for the remaining six). The remaining 12 samples were commercialised as bark (11 *C. verum* and 1 cassia).

To evaluate if multivariate analysis of the elemental profiles was able to detect adulteration of *C. verum* with other cinnamon varieties, some samples labelled as *C. verum* were randomly selected and mixed with different amounts of samples labelled as *C. burmanii*, *C. aromaticum*, or cassia. The final mixtures contained 5, 10, 20, 30, 40, 50, and 60% of the cinnamon belonging to the “cassia” group.

### Reagents and standards

Deionised water obtained with a Milli-Q Plus system (> 18.3 MΩ) (Millipore, Billerica, MA, USA) was used to clean the sample processing material to avoid cross-contamination between samples.

The reference sample FLX-S13 (Fluxana, Bedburg-Hau, Germany) was used to check the performance of the EDXRF instrument.

Forty-five certified reference materials (CRMs) and reference materials (RMs), 23 of them with an organic matrix, were used to construct calibration curves for the determination of macro and trace elements by EDXRF. The organic CRMs and RMs used to construct calibration curves were ERM-CD-281 (ray grass), NBS 1571 (orchard leaves), NBS1572 (citrus leaves), BCR-129 (hay powder), NIST 1570a (spinach leaves), NIST 1575a (pine needles), ERM-CD-200, NMIJ CRM7405-a, IAEA-392, IAEA-413 (algae), NIST 1567b (wheat flour), BCR-482, IAEA-336 (lichen), OBTL-5 (tobacco), GSV1 and GSV2 (bush leaves), GSV3 (poplar leaves), GSV4 (tea leaves), GSH1 (human hair), BCR-679 (white cabbage), and IAEA-359 (cabbage).

The accuracy of the method was evaluated with 25 CRMs and RMs, 13 of them with organic matrix. The organic CRMs and RMs were NIST 1568b (rice flour), BCR-191 (brown bread), SOWW-1 (soft winter wheat flour), DUWF-1 (durum wheat flour), BRAN-1 (corn bran), PVTL-6, RT3, RT5, AJJA17 (tobacco), ERM-BD150 (skimmed milk), ERM-CE 2781 K (mussel tissue), ERM-CE464 (tuna fish), and IMEP-119 (vegetable feed).

### Sample preparation

*C. verum* and cassia cinnamon samples purchased as bark were milled with a Planetary MonoMill-Minimill II of Fritsch-PANalytical (Almero, The Netherlands) at 300 rpm for 6 and 10 min, respectively. The mill consisted of a 250 tungsten carbide bowl and 3 grinding ball of the same material with a diameter of 20 mm. To carry out the measurements by EDXRF, the fine powder obtained was used to make 40-mm-diameter pellets using aluminium cup dies and pressing the sample with a semi-automatic press of Hertzog Maschinenfabrik Gmbh (Osnabrück, Germany) at 250 kN for 3.5 min. The samples that were sold already ground were thoroughly mixed with a metal-free spatula before proceeding to the preparation of the 40-mm pellets, as described earlier.

### Instrumentation

#### Elemental analysis by EDXRF

The elemental composition of the cinnamon samples was determined by EDXRF, using an Epsilon 5 (PANalytical, Almelo, The Netherlands) spectrometer and a method previously optimised and validated for the analysis of organic and inorganic matrices, using the CRMs and RMs listed above. Detailed information about accuracy, working range, limit of quantification (LoQ), and expanded uncertainties for the different elements can be found elsewhere [23].

The performance of the EDXRF spectrometer was checked weekly with the reference sample FLX-S13 (Fluxana, Bedburg Hau, Germany), following the instructions of the manufacturer. The instrument was calibrated every week with the mentioned reference sample correcting in this way the normal drift of the instrument. No systematic bias was observed during the period that the measurements lasted.

#### Volatile analysis by HS-GC–MS

A cinnamon mix containing 1 g of each of the 52 cinnamon samples was prepared for method development. The mixed sample was thoroughly homogenized and aliquots of 25 ± 1 mg were weighed into 22-mL crimp vials. The samples were spiked with 5 µL of internal standard solution (2-methoxy-4-propyl phenol, 0.5 mg mL^−1^ in methanol) and closed with aluminium crimp seals with PTFE-coated silicone septa. Headspace analyses were performed with a TurboMatrix 110 Trap headspace sampler (PerkinElmer). The tested conditions of vial equilibration temperature and time for headspace sampling were 70, 80, 100, 125, and 150 °C and 15 and 30 min, respectively. The combination of 150 °C and 15 min was chosen for all the cassia and Ceylon cinnamon samples because they were sufficient to detect the compounds of interest in *C. verum* and cassia samples. No further optimisation was carried out since it would go beyond the main scope of this study. Volatile compounds were sampled with the syringe needle at 135 °C, pre-concentrated in the instrument trap held at 30 °C, desorbed at 280 °C, and transferred to an Agilent 7890B/7010 GC–MS with the transfer line at 200 °C. Sample injection was performed with a split ratio of 10:1 and the inlet at 180 °C. Compound separation was performed on a DB-5MS capillary column (30-m length, 0.25-mm inner diameter, 0.25-μm film thickness) with helium carrier gas at 1 ml L^−1^. The GC oven temperature was started at 40 °C for 5 min, then ramped at 5 °C min^−1^ to 300 °C. Mass spectra were acquired with electron ionisation (70 eV) over the 30–700 m/z range in total ion current mode. Structural assignments were based on searching against the NIST mass spectral library version 2.0 2011 [24]. Semi-quantitative analysis was performed by calculating the normalized peak area of detected volatile compounds per gram of cinnamon (NA) taking into account compound peak area (A_*x*_), internal standard peak area (A_*is*_), internal standard quantity (q_*is*_ in µg), and cinnamon weight (*q*_cinnamon_ in g):

$$NA={A}_{x}{\cdot A}_{is}^{-1} \cdot {q}_{is}\cdot {q}_{\mathrm{cinnamon}}^{-1}$$

#### Ash determination by TGA

The ash content of the samples was determined with a thermogravimetric analyzer (Mettler Toledo). Test materials were measured in duplicate with test portion sizes ranging from 31 to 64 mg. The test portions were heated in an oxidative atmosphere to 550 °C and held at that temperature until the weight of the residue stayed constant. The mass % of the residue was reported as the ash content.

### Multivariate analysis

Multivariate analysis of the elemental and volatile mass fractions of the cinnamon samples was carried out with the software SIMCA® version 17 (Umetrics, Malmö, Sweden) [25]. Only the elements whose mass fractions were significantly different for *C. verum* and cassia cinnamon (Student’s test, 95% confidence interval) were used for modelling purposes and are Al, Si, P, Cl, S, K, Ti, Fe, Ni, Cu, Zr, Se, and Mo. Mass fractions below the LOQ were also used for modelling purposes because even if below the LOQ some of them were above the LOD and small differences could be detected. The Student’s tests were run using the software Statistica version 13.0.5.17 (TIBCO, Software Inc.).

Principal component analysis (PCA), a non-supervised multivariate analysis tool that reduces the amount of variables (elemental and volatile mass fractions) into a reduced number of non-correlated principal components, was used to visualise clusters in function of their botanical variety. Concentrations were normalised by unit-variance scaling. No further pre-processing was applied. The amount of principal components was always set to three to avoid overfitting.

## Results and discussion

### Detection of anomalous samples based on the elemental profiles determined by EDXRF

The following elements—Mg, Al, Si, P, Cl, S, K, Ca, Ti, Cr, Mn, Fe, Ni, Cu, Zn, As, Br, Rb, Sr, Zr, Nb, Cd, Ba, Pb, Hg, V, Co, Se, Mo, Sn, Sb, Cs, La, Sm, Ce, and Nd—were analysed in all the samples included in the study. Not all the elements could be quantified but some of the non-quantifiable elements could be detected in some samples. The more abundant elements in both species were K and Ca. In general, the elemental content of cassia samples was lower than that of Ceylon cinnamon, Supplementary 1, Mn being the only element that was significantly more abundant in cassia than in Ceylon cinnamon.

**Fig. 1:**
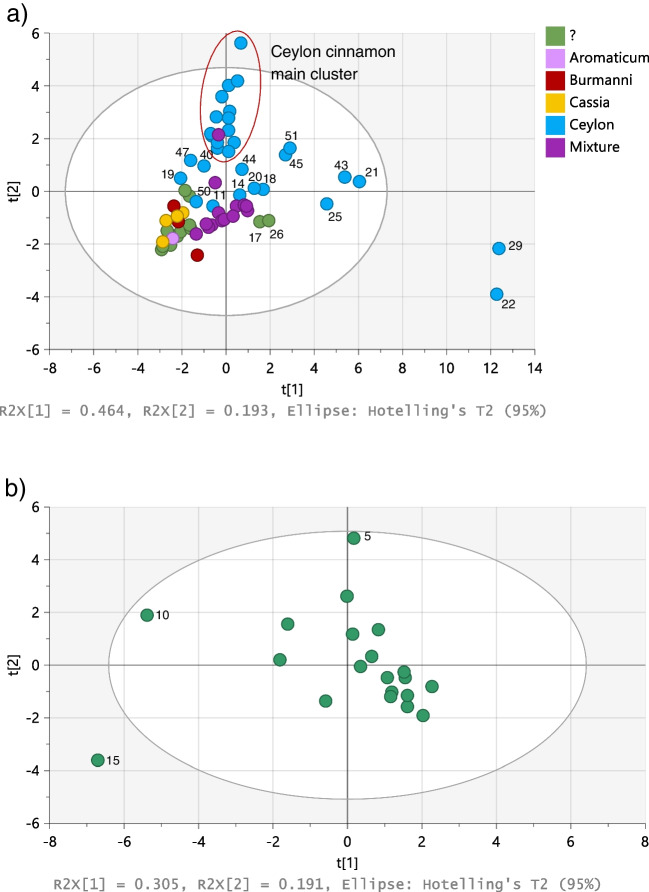
PCA score plots obtained with the elemental profile of (**a**) Ceylon and cassia cinnamon, and (**b**) cassia cinnamon samples analysed by EDXRF

Figure 1a shows the PCA score plot of all the samples analysed, coloured according to the botanical variety indicated on the label. This plot was constructed using exclusively the variables that, according to the Student’s *t*-test, are significantly different in the cassia and Ceylon cinnamon groups (Al, Si, P, Cl, S, K, Ti, Fe, Ni, Cu, Zr, Nb, V, Co, Se, Mo, Cs, and Nd). Figure 1a included a set of samples prepared in our laboratory, mixing different amounts of Ceylon and cassia cinnamon samples, randomly selected, so that the final mixtures contained 5, 10, 20, 30, 40, 50, and 60% of cassia. The mixture samples were used to elucidate if some of the samples included in the study could be a mixture of Ceylon and cassia cinnamon. One “mixture” sample containing 20% cassia cinnamon is projected among the Ceylon cinnamon main cluster in the score plot showed in Fig. 1a, constructed with the two first principal components (PCs). Nevertheless, that sample is separated from the main Ceylon cinnamon cluster along the third PC.

As expected, the samples labelled as “*Burmanii*”, “*Aromaticum*”, and “C*assia*” cluster together in the model, as well as most of the samples without label indication on botanical variety. The latter (marked as “?” in Fig. 1a) were accordingly included in the “cassia” group for further studies. Two samples without information about botanical variety, 17 and 26, were projected separated from the cassia group. Sample 17 was according to the label, a mixture of cinnamon from Sri Lanka and Indonesia, and could therefore be a mixture of cassia and Ceylon cinnamon. No information is provided about the botanical variety of sample 26 but, according to the label, the sample was produced in Vietnam and could be a *C. loureiroi.* However, this fact only would not explain the sample to be projected separated from the cassia group because sample 24, also a cassia sample produced in Vietnam, is projected together with the main cluster of cassia samples. Samples 17 and 26 were projected next to the “mixture” group, what would confirm that both samples are a mixture of cassia and Ceylon cinnamon. Four more samples labelled as Ceylon cinnamon were also projected either together with the “mixture” samples (11 and 14) or next to them (18 and 20); sample 44 was projected between the “mixture” group and the main Ceylon cinnamon cluster. Four more Ceylon cinnamon labelled samples, 19, 40, 47, and 50, were projected together with the cassia samples. Since the nine samples were labelled as Ceylon cinnamon, they could represent cases of fraud by total or partial substitution of Ceylon cinnamon with cassia. Nevertheless, further studies using other analytical approaches had to be carried out in order to confirm that suspicion.

It can also be seen in Fig. 1a that five Ceylon cinnamon samples (21, 22, 25, 29, and 43) are plotted separated from the rest of the samples; in particular, samples 22 and 29 are plotted outside the Hotelling’s T2 (95%) ellipse, three times outside the standard deviation of the model. The five samples are characterised by having significantly higher mass fractions of Al, Si, Ti, Fe, Ni, and Zr, than the rest of the samples all together (Ceylon as well as cassia cinnamon). The mass fractions of Al, Si, and Ti, in some of the mentioned samples, are lower than the limit of quantification of the method (860, 2348, and 302 mg kg^−1^, respectively), but still clearly detectable and higher than in the remaining samples. Samples 21, 22, and 29 were sold in bulk. It has already been published in a study on assessment of health risk due to toxic elements present in cinnamon that the highest Fe and Al contents were found in bulk cinnamon samples [15].

Particularly interesting are the high contents of Zr in the five mentioned samples, in the range 9.64–24.39 mg kg^−1^ (LOQ 10 mg kg^−1^), keeping in mind that the Zr levels in food normally fall in the range 0.005–2.6 mg kg^−1^ [26]. Samples 11, 14, 17, 18, 20, 26, 45, 49, and 51 also had Zr mass fractions equal or higher than 2.6 mg kg^−1^, but lower than 10 mg kg^−1^, LOQ of the method used. The Zr content in all the samples purchased as bark was below 1 mg kg^−1^. Concentrations lower than the LOQ of our method can only be considered as indicative of higher or lower content in the relevant samples.

The relatively high contents of Al, Si, Ti, Fe, and Zr in the mentioned samples could be due to the presence of some extraneous substances, even of inorganic nature. A way to test this hypothesis would be to determine the total ash content of the samples.

### Analysis of total ash by TGA

ISO 6539 [27] and ISO 6538 [28] specify requirements for C. *verum* and cassia, respectively, but do not address fraudulent manipulations of any of the two spices. One of the parameters covered is the maximum total ash content. ISO 6539 sets a maximum total ash content in Ceylon cinnamon of 5% (mass fraction) in Sri Lankan type, and of 7% in Seychelles and Madagascan type. Maximum limits for total ash content in cassia are 4.0% in Chinese type, 4.5% in Vietnamese type, and 5.0% in Indonesian type (ISO 6538).

Total ash contents in the studied samples as obtained by TGA are given in Table 1. The total ash content of samples 21, 22, 25, 29, and 43 was higher than 5% (maximum allowed total ash content for Sri Lanka cinnamon type) but lower than 7% (maximum allowed total ash content for Madagascan or Seychelles cinnamon type). No information about the geographical origin of those samples was given on the respective labels. The samples would fulfil the ISO 6539 quality criteria for total ash if they were produced in Madagascar or Seychelles.

Samples 18, 20, 45, and 51 are slightly above or under 5% total ash content. The total ash content in sample 26 is 4.4 ± 0.7%, which is around the maximum limit of 4.5% set in ISO 6538 for Vietnamese cassia. The position of samples 18, 20, 21, 22, 25, 29, 43, 45, and 51 in the PCA score plot constructed with the elemental profiles, Fig. 1a, can be explained by the high ash content of those samples.

The total ash content of samples 11, 14, and 17 was below the maximum limit set by ISO 6539, certainly keeping in mind that samples 11 and 14 were produced in Madagascar according to their respective labels. Further analyses have to be carried out to explain their projection in Fig. 1a.

Two samples in the cassia group (5 and 16) had a total ash content higher than the maximum set in ISO 6538. Sample 16 is according to the label *C. aromaticum*, and hence the maximum ash content taken as reference was 4%. Sample 10 was the cassia sample with the lowest total ash content, 3.2%. The PCA score plot constructed with the elemental profile obtained by EDXRF for the cassia samples, Fig. 1b, shows that samples 5 and 10 are indeed projected separated from the main cluster. Sample 15 is also projected separated from the main cluster mostly due to a high Fe mass fraction, the second highest in the cassia group after sample 17, probably a mixture of cassia and Ceylon cinnamon.

The total ash content in the 12 samples purchased as bark covered the range 4.1 to 6.5%, with seven samples having a total ash content higher than 5%. Five out of the seven samples were produced in Sri Lanka and do not comply with the ISO 6539 quality criteria. No information about geographical origin was available for the remaining two samples.

Contrary to ground samples, bark samples with a total ash content higher than 5% do not have significantly higher Al, Si, Ti, Fe, Ni, and Zr mass fractions. In bark samples, the main contributors to the increased total ash were K and/or Ca. The high total ash content in those samples could be due to the presence of extraneous substances, including parts of the plant other than bark, rolled inside the external bark layer, or to some intrinsic characteristic of the samples (e.g., bark thickness, plant age).

**Fig. 2:**
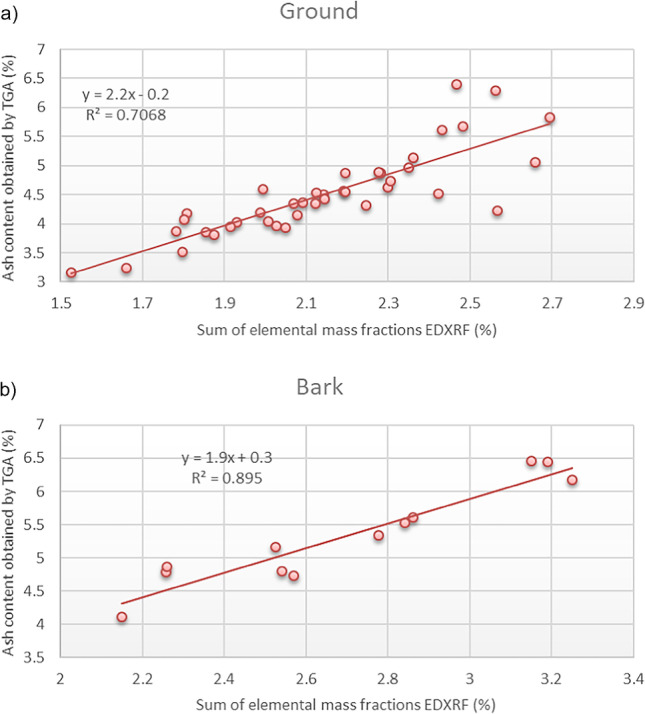
Calibration curves correlating the ash content determined by TGA and the mass fractions of the main elements (Mg, Al, Si, P, Cl, K, Ca, Ti, Mn, Fe, Ni, Cu, and Zn) in (**a**) ground and (**b**) bark cinnamon samples

The sum of Mg, Al, Si, P, Cl, K, Ca, Ti, Mn, Fe, Ni, Cu, and Zn mass fractions is directly correlated with the total ash content calculated by TGA, both in ground and bark samples, as shown in Fig. 2a and b, respectively. Making use of those calibration curves, samples 5, 33, 35, 36, 37, 38, 39, and 42 were also flagged as suspicious based on EDXRF results. Only sample 16 was not flagged as suspicious of a high ash content based on the EDXRF results. The elemental mass fractions obtained by EDXRF can be used to flag samples suspicious of having a total ash content higher than the maximum limits stablished by ISO standards. The number of samples needing to undergo analysis for ash content would be reduced in this way, saving time and money to control laboratories.

### Detection of anomalous samples based on semi-quantitative analysis of the volatile composition by HS-GC–MS

It is well known that Ceylon cinnamon and cassia cinnamon have a different profile of volatiles [9]. Ceylon cinnamon can be identified by the presence of eugenol and benzyl benzoate, both absent in cassia, while coumarin and δ-cadinene, present in cassia, are either absent or present in small amounts in Ceylon cinnamon.

For this reason, the conclusions on suspicious/non-suspicious samples based on elemental profiles were cross-checked by semi-quantitative HS-CG-MS analyses of the volatile composition of all samples in the study.

Seventy-six substances were identified in all or some of the samples analysed, Supplementary 2. As expected, the compound most abundantly present in both Ceylon and cassia cinnamon was cinnamaldehyde, while coumarin and δ-cadinene were clearly more abundant in cassia. Only traces of the latter compounds could be found in Ceylon cinnamon. Eugenol and benzyl benzoate were absent in almost all cassia samples, with only traces of eugenol found in samples 2 and 24. The same brand that commercialises sample 24 commercialises also Ceylan cinnamon, and cross-contamination during production cannot be excluded.

**Fig. 3:**
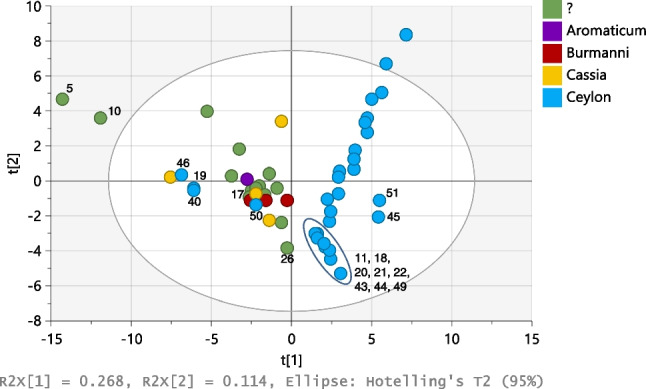
PCA score plot obtained with the volatile profile of Ceylon and cassia cinnamon samples analysed by semi-quantitative HS-GC–MS

The normalised areas of volatile compounds obtained (semi-quantitative) were used to construct the PCA score plot shown in Fig. 3. Two clearly separated clusters corresponding to the Ceylon cinnamon and the cassia groups, respectively, are observed. The volatile composition did not allow differentiating the varieties within the “cassia” group. Most of the samples without label indication on botanical variety (marked as “?” in Fig. 3) clustered together with the “cassia” samples, confirming the outcome of EDXRF analyses.

The score plot shows that four samples labelled as Ceylon cinnamon (19, 40, 46, and 50) were projected together with the cassia group. Indeed, neither eugenol nor benzyl benzoate, both markers of Ceylon cinnamon, was detected in samples 19, 40, and 50. Sample 46 does not contain detectable amounts of eugenol but it contained traces of benzyl benzoate, suggesting that the sample could contain a small amount of Ceylon cinnamon. These results confirm the findings previously obtained by EDXRF, according to which samples 19, 40, and 50 were suspicious of being cassia or a mixture cassia-Ceylon cinnamon. However, sample 46 was not suspicious of being totally or partially a cassia sample based on its elemental profile and would have been a false negative if only EDXRF analyses would have been carried out.

The results obtained by EDXRF suggested that samples 17 and 26 could be a mixture of cassia and Ceylon cinnamon. This hypothesis was confirmed for sample 17 by HS-GC–MS analysis, because it contained coumarin and δ-cadinene at concentrations that fall in the range covered by cassia samples, while also containing benzyl benzoate and eugenol, markers of Ceylon cinnamon. Sample 26 contains coumarin and δ-cadinene but it does not contain neither benzyl benzoate nor eugenol, in detectable amounts. However, sample 26 is of all the samples analysed the one with the lowest content of cinnamaldehyde (about 60% the average of the other samples). This could indicate that the sample is a mixture of cassia with some other material, maybe inorganic, what would explain the Al, Si, Ti, Fe, and Zr mass fractions, higher in this sample than in the average of the cassia samples. This explanation would also be supported by its ash content, 4.4 ± 0.7%, just around the maximum limit for total ash given in ISO 6538 for Vietnamese cassia. Sample 26 comes from Vietnam and this could also explain its projection in Figs. 1a and 3, separated from the rest of the Ceylon and cassia cinnamon samples. More samples from Vietnam should be analysed to come up with a clear conclusion on sample 26.

Samples 45 and 51 are plotted separated from the main cluster of Ceylon cinnamon samples, supporting the information provided by EDXRF analysis for the same two samples. Figure 3 also shows a compact sub-cluster within the Ceylon cinnamon cluster that groups samples 11, 18, 20, 21, 22, 43, 44, and 49. All the samples in that sub-cluster were also pointed as suspicious based on the results of the EDXRF analysis. Samples 21, 22, 25, 29, 44, 45, 49, and 51 were together with sample 14, the only Ceylon cinnamon samples in which 2-bornanone (camphor) was detected. Camphor was also present in sample 17, mixture of Ceylon cinnamon and cassia. Camphor is the main volatile compound (60%) in the cinnamon root, and this finding suggests that in the mentioned samples bark had been partially substituted by cinnamon root. This would explain the relatively high contents of Al, Si, Ti, Fe, Ni, and Zr found in samples 21, 22, 25, and 29, and the relatively high contents of Zr in samples 14, 17, 45, 49, and 51 that suggested the presence of some inorganic material in the samples. The partial substitution of bark by other plant material in those samples would also explain the lower amount of some of the volatile compounds present in bark, as it is the case of hydrocinnamaldehyde [29] whose content in samples 21, 22, 25, 29, 43, 45, and 51 is around one-third the average content in the other Ceylon cinnamon samples. Some of the samples in the mentioned sub-cluster, samples 11, 18, 20, 29, 43, 45, 49, and 51, have higher contents of methyl esters of some fatty acids such as myristic, palmitic, linoleic, oleic, and stearic acids. Methyl esters of these fatty acids could be formed by partial methylation of the free acids by the internal standard solution containing methanol during the extraction of volatiles that occurred at 150 °C for 15 min. This possibly explains why the mentioned fatty acids, if present in the sample, could be detected by HS-GC–MS without any derivatisation step in the sample preparation. Seeds are rich in fatty acids [30] and the presence of detectable amounts of some fatty acids in the mentioned samples would suggest that they contain not only cinnamon bark but also seeds.

Among the bark Ceylon cinnamon samples with a total ash content higher than 5%, samples 33, 35, 36, 37, 39, and 42 had a significantly higher content of benzyl alcohol and p-cymenene (two to three times higher), and a significantly lower content of α-pinene, camphene, and d-limonene (around three times lower) than the remaining *C. verum* bark samples. Cinnamon bark could have been partially substituted by other plant material, including other parts of the cinnamon plant. For instance, the eugenol content in sample 39 is about six times higher than the average of all the other Ceylon cinnamon samples purchased as bark, not ground. It is known that eugenol is the most abundant volatile compounds in Ceylon cinnamon leaves, 70 to 95% in comparison with the 5 to 10% in bark [8]. Sample 38 contains seven times more caryophyllene oxide (found in Ceylon cinnamon flowers) than the average of the samples purchased as bark [8].

Sample 47, also purchased as bark, was according to EDXRF analysis suspicious of being a cassia sample or a mixture Ceylon cinnamon with cassia. However, the volatile composition of sample 47 did not support that suspicion because it only contains traces of coumarin and δ-cadinene, while being the Ceylon cinnamon sample with the highest content of benzyl benzoate. Its content in eugenol is also higher than the average in Ceylon cinnamon samples. Sample 47 is significantly different from the other Ceylon cinnamon in its high content of cinnamyl acetate, predominant compound in fruit, flowers, and fruit stalks of Ceylon cinnamon [7]. Sample 47 could contain parts of the cinnamon plant different from the bark, what would explain its projection in the elemental PCA score plot, Fig. 1a, separated from the main cluster of Ceylon cinnamon samples.

Regarding the cassia samples, Fig. 3 shows that samples 5 and 10 are projected separated from the rest of the cassia samples, confirming the results obtained by EDXRF and TGA. Both samples are richer than the other cassia samples in a number of volatile compounds; in particular, they were the richest among the cassia samples in ylangene, cyclosativene, α-copaene, isosativene, γ-muurolene, calamenene, α-calacorene, and gleenol.

According to all the previous discussion, only one sample, sample 46, labelled as Ceylon cinnamon but identified as cassia cinnamon by its volatile composition, would not have been flagged as suspicious by EDXRF (false negative). All the samples considered as suspicious by their elemental profiles were also suspicious by HS-GC–MS and/or TGA (zero false positives); hence, the accuracy of the EDXRF method in the detection of fraudulent Ceylon cinnamon is 98% [31].

While there is a good agreement between the conclusions extracted from the EDXRF and the TGA results, not all the samples flagged as suspicious by their elemental profiles and by their ash content determined by TGA were suspicious on the basis of their volatile composition. The ash content in food refers to the mineral fraction left after moisture, volatile fraction, and organic fraction have been removed by heating the sample up to very high temperatures; therefore, the agreement between EDXRF and TGA is to be expected. The correlation between TGA and the results obtained by GC–MS is not so obvious. Samples declared suspicious by GC–MS could be compliant with the ash content if the material used to replace the *C*. *verum* bark has the same or similar ash content as the bark. This could be the case of samples 44, 45, and 47. A high ash content is not necessarily reflected in the volatile composition of the sample and it could be the result of just poor quality of the product, as discussed in the “Analysis of total ash by TGA” section. Samples such as 33, 35, 36, 37, 39, and 42 have some peculiarities in their volatile composition as described above; nevertheless, since no clear link could be stablished between those features and a specific type of fraudulent manipulation, they were not flagged as suspicious in Table 1.

**Fig. 4:**
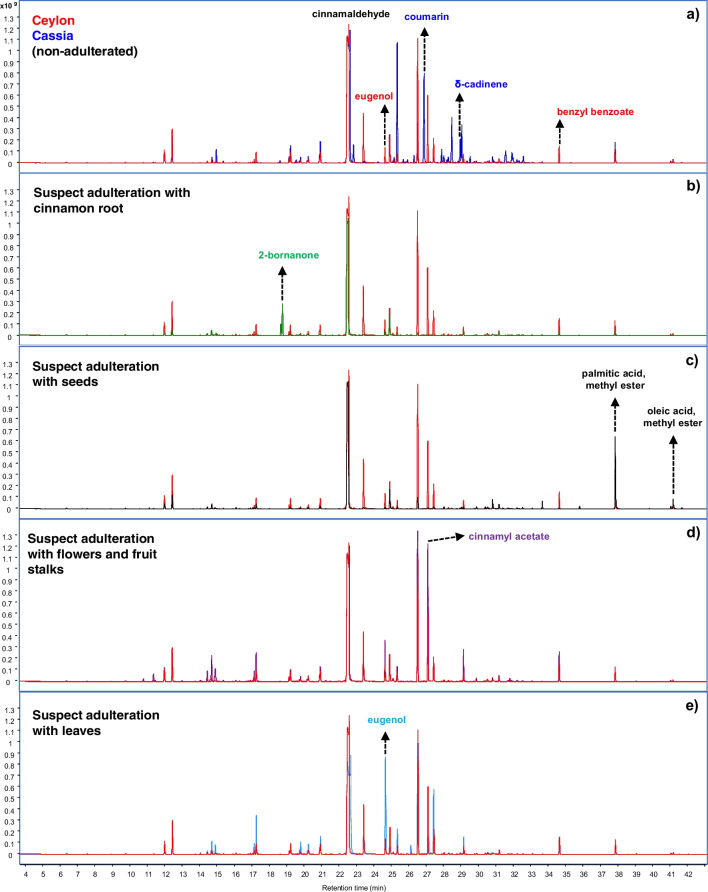
Overlay of chromatograms obtained by HS-GC–MS for pure Ceylon cinnamon samples (red) with: (**a**) pure cassia (blue), (**b**) Ceylon cinnamon with cinnamon root (green), (**c**) Ceylon cinnamon with seeds (black), (**d**) Ceylon cinnamon with flowers and fruit stalks (magenta), (**e**) Ceylon cinnamon with leaves, showing the compounds that were indicative of the respective adulteration

Figure 4 provides chromatograms obtained by HS-GC–MS, showing the compounds that pointed at some type of adulteration.

## Conclusions

Ceylon cinnamon should be regularly included in control plans to detect fraud since a high rate of commercially available products are either fraudulent or do not fulfil some international quality requirements. Fifty-eight percent of the samples included in the study were either suspicious of some type of adulteration, including substitution of Ceylon with cassia cinnamon or some inorganic extender, and substitution of bark with some other part of the cinnamon plant, or did not respect international standards on total ash content. Four out of the 29 Ceylon cinnamon samples analysed were either pure cassia cinnamon or a mixture containing that spice.

The very high rate of adulteration in commercially available Ceylon cinnamon is the result of the lack of a standardised method, and of the difficulty in carrying out GC–MS analyses and in the interpretation of the results obtained with that approach. A method easy to implement in control laboratories is needed.

The outcome of the study confirmed the initial hypothesis that the elemental profiles obtained by EDXRF can be used for screening purposes to detect irregularities in Ceylon cinnamon samples. This approach would reduce the number of samples that need to be characterised for their volatile composition and/or total ash content. The infrastructure to carry out elemental analysis is widely available throughout the world and techniques such as ICP-MS or ICP-AES can also be used.

No single element can be used to detect fraudulent practices in Ceylon cinnamon products, and multivariate analyses are needed. However, more and more laboratories are knowledgeable in multivariate data treatment and modelling that can be carried out with software available for free. However, control laboratories that would like to use elemental profiles for authentication purposes would need to train their models with *C*. *verum* and cassia samples with a full traceability record, on which no doubts about their purity would exist. The creation of a central database with elemental profiles of reference Ceylon cinnamon and cassia samples from different geographical origin, to which control laboratories could have access, would facilitate control activities.

## Supplementary Information

Below is the link to the electronic supplementary material.

Supplementary file1 (DOCX 33 KB)

Supplementary file2 (DOCX 57 KB)
