# Supplementary material for: Use of elemental profiles determined by energy-dispersive X-ray fluorescence and multivariate analyses to detect adulteration in Ceylon cinnamon
Source: Anal Bioanal Chem. 2023 Aug 17;415(22):5437–49. doi: 10.1007/s00216-023-04817-1 (PMC10444698; doi:10.1007/s00216-023-04817-1)
Supplement: Supplementary file 1 — Supplementary file1 (DOCX 33 KB) [file 216_2023_4817_MOESM1_ESM.docx]

**Supplementary 1:** Mass fractions of major and trace elements in the analysed cassia and Ceylon cinnamon samples (all values expressed in

mg kg^-1^). **a)** Mg, Al, Si, P, Cl, S, K, Ca, Ti, Cr, Mn, Fe, Ni, **b)** Cu, Zn, As, Br, Rb, Sr, Zr, Cd, Ba, Pb, Hg, Se, Mo.

**a)**

| **Sample id** | **Label variety** | **Origin** | **Mg LOQ 1450**  **mg kg^-1^**  **U(k=2)**  **13 %** | **Al LOQ**  **860**  **mg kg^-1^**  **U(k=2)**  **17 %** | **Si LOQ 2348**  **mg kg^-1^**  **U(k=2)**  **12 %** | **P LOQ**  **171**  **mg kg^-1^**  **U(k=2)**  **6 %** | **Cl LOQ**  **78**  **mg kg^-1^**  **U(k=2)**  **2 %** | **S LOQ**  **700**  **mg kg^-1^**  **U(k=2)**  **10 %** | **K LOQ**  **566**  **mg kg^-1^**  **U(k=2)**  **3 %** | **Ca LOQ**  **118**  **mg kg^-1^**  **U(k=2)**  **3.5 %** | **Ti LOQ**  **302**  **mg kg^-1^**  **U(k=2)**  **10 %** | **Cr LOQ**  **1.89**  **mg kg^-1^**  **U(k=2)**  **20 %** | **Mn LOQ**  **2.55**  **mg kg^-1^**  **U(k=2)**  **11 %** | **Fe LOQ**  **4.60**  **mg kg^-1^**  **U(k=2)**  **6.5 %** | **Ni LOQ**  **0.16**  **mg kg^-1^**  **U(k=2)**  **25 %** |
| --- | --- | --- | --- | --- | --- | --- | --- | --- | --- | --- | --- | --- | --- | --- | --- |
| 1 | NA | NA | 1889 | ND | *62.6* | 380 | 115 | *662* | 5517 | 12266 | ND | *1.42* | 187 | 56.2 | *0.14* |
| 2 | NA | NA | 1806 | ND | *313* | 336 | 139 | *632* | 5597 | 13368 | ND | *1.47* | 208 | 131 | *0.11* |
| 3 | NA | NA | 1649 | ND | *97.4* | 343 | 154 | *668* | 5236 | 11523 | ND | *1.40* | 232 | 47.1 | *0.11* |
| 4 | NA | NA | 1796 | ND | *12.1* | 258 | 95.7 | *599* | 5211 | 13760 | ND | *0.90* | 213 | 53.4 | *0.10* |
| 5 | NA | NA | 2792 | *31.8* | ND | 431 | 80.8 | 1137 | 7045 | 15752 | ND | *1.53* | 413 | 24.4 | 0.17 |
| 6 | NA | NA | 1928 | ND | *61.8* | 236 | *36.2* | *616* | 4808 | 14670 | ND | *1.11* | 176 | 22.0 | *0.13* |
| 7 | NA | NA | 2145 | ND | *44.6* | 313 | 129 | 705 | 5406 | 12528 | ND | *1.34* | 170 | 34.5 | *0.07* |
| 8 | NA | NA | 2529 | ND | *30.5* | 258 | *72.9* | *591* | 4618 | 15231 | ND | *1.22* | 206 | 23.6 | *0.15* |
| 9 | NA | NA | 1856 | ND | *35.1* | 339 | 91.0 | *676* | 5136 | 12552 | ND | *1.48* | 209 | 32.2 | *0.02* |
| 10 | NA | NA | *1069* | ND | *244* | 295 | *35.5* | 1150 | 4862 | 8053 | ND | 1.97 | 542 | 137 | 0.43 |
| 11 | Ceylon | Madagascar | 1878 | ND | *68.4* | 385 | 572 | 838 | 7530 | 9003 | ND | *1.21* | 315 | 295 | 0.66 |
| 12 | Ceylon | Sri Lanka | 1557 | ND | *332* | 1139 | 644 | 7205 | 9028 | 12537 | ND | *1.24* | 112 | 290 | 0.44 |
| 13 | NA | NA | 1729 | ND | *132* | 366 | 147 | *638* | 5946 | 14739 | ND | *1.47* | 244 | 182 | *0.12* |
| 14 | Ceylon | Madagascar | 1709 | ND | *365* | 390 | 491 | 749 | 7560 | 13073 | *3.18* | *1.80* | 239 | 371 | 0.37 |
| 15 | Burmanni | NA | 1916 | ND | *346* | 271 | 107 | *628* | 5428 | 12645 | ND | *1.08* | 198 | 322 | 0.18 |
| 16 | Aromaticum | NA | 1845 | ND | *172* | 358 | 108 | *636* | 5310 | 12638 | ND | *1.35* | 187 | 45.3 | *0.02* |
| 17 | NA | Ceylan and  Indonesia | 1771 | *56.7* | *412* | 349 | 261 | 1018 | 6799 | 10686 | *17.8* | 3.78 | 279 | 571 | 1.13 |
| 18 | Ceylon | NA | 1464 | *16.2* | *398* | 462 | 374 | 896 | 9098 | 10912 | *6.09* | *1.88* | 190 | 671 | 0.30 |
| 19 | Ceylon | NA | *1234* | ND | *138* | 609 | *53.7* | 3914 | 4835 | 9351 | ND | *1.22* | 307 | 66.3 | 0.23 |
| 20 | Ceylon | NA | 2157 | ND | *457* | 428 | 311 | 794 | 8342 | 9473 | ND | *1.53* | 195 | 566 | 0.29 |
| 21 | Ceylon | NA | 1763 | *313* | *954* | 566 | 532 | 3492 | 8274 | 11744 | *61.4* | 10.8 | 218 | 1175 | 2.35 |
| 22 | Ceylon | NA | 2424 | 1629 | *925* | 300 | 676 | 846 | 6624 | 9457 | *162* | 15.0 | 215 | 1881 | 4.69 |
| 23 | Cassia | NA | 1910 | ND | ND | 356 | 183 | *654* | 5517 | 12753 | ND | *1.62* | 153 | 29.2 | *0.14* |
| 24 | Cassia | Vietnam | 1653 | ND | *176* | 392 | *70.3* | 852 | 6126 | 11059 | ND | *0.91* | 323 | 68.4 | 0.16 |
| 25 | Ceylon | NA | 2011 | *76.5* | *757* | 598 | 749 | 1640 | 8645 | 10881 | *39.7* | 2.55 | 126 | 906 | 0.46 |
| 26 | NA | Vietnam | 1793 | *196* | *439* | 396 | 84.3 | 1776 | 6509 | 10903 | *13.1* | *1.67* | 335 | 756 | 0.55 |
| 27 | Burmanni | NA | 1596 | ND | *143* | 393 | *61.5* | 749 | 5037 | 10537 | ND | *0.91* | 231 | 56.8 | 0.20 |
| 28 | Burmanni | NA | 1795 | ND | *151* | 351 | 94.9 | *537* | 4888 | 12415 | ND | *0.87* | 161 | 61.8 | 0.25 |
| 29 | Ceylon | NA | 2761 | 1943 | *1405* | 363 | 666 | 1014 | 8094 | 7128 | *166* | 6.85 | 218 | 1881 | 1.99 |
| 30 | Cassia | NA | *1397* | ND | ND | 369 | *56.1* | *569* | 4829 | 11159 | ND | *0.91* | 159 | 37.9 | 0.26 |
| 31 | NA | Sri Lanka | 1815 | ND | ND | 474 | 231 | 750 | 5514 | 10817 | ND | *1.23* | 202 | 66.8 | 0.19 |
| 32 | Ceylon | Sri Lanka | 2538 | ND | *40.4* | 545 | 373 | 1131 | 8791 | 13274 | ND | *1.24* | 67.5 | 43.4 | 0.18 |
| 33 | Ceylon | Sri Lanka | 2236 | ND | ND | 1131 | 381 | 5251 | 9573 | 15099 | ND | 2.29 | 133 | 37.9 | 0.39 |
| 34 | Ceylon | Sri Lanka | 1497 | ND | *123* | 682 | 368 | 3848 | 6834 | 12808 | ND | *1.25* | 165 | 68.1 | 0.24 |
| 35 | Ceylon | Sri Lanka | 2934 | ND | ND | 677 | 725 | 931 | 9228 | 18864 | ND | *1.68* | 31.7 | 21.3 | 0.43 |
| 36 | Ceylon | Sri Lanka | 2684 | *4.57* | ND | 482 | 493 | *638* | 7948 | 19770 | ND | *1.29* | 70.9 | 25.8 | 0.46 |
| 37 | Ceylon | NA | 2131 | ND | ND | 655 | 508 | 1157 | 9966 | 14313 | ND | *1.26* | 108 | 61.0 | 0.31 |
| 38 |  | NA | 1648 | ND | *90.6* | 376 | 786 | 819 | 8864 | 13309 | ND | *1.74* | 198 | 23.4 | 0.25 |
| 39 | Ceylon | Sri Lanka | 2222 | *85.3* | ND | 805 | 502 | 4198 | 10983 | 17168 | ND | *0.97* | 127 | 40.0 | 0.45 |
| 40 | Ceylon | NA | *1140* | ND | *305* | 568 | *41.2* | 3500 | 5976 | 9957 | ND | *1.06* | 382 | 164 | 0.35 |
| 41 | Ceylon | Sri Lanka | 2123 | ND | *220* | 701 | 446 | 1331 | 10571 | 7919 | ND | *1.42* | 231 | 231 | 0.42 |
| 42 | Ceylon | Sri Lanka | 2221 | ND | *35.5* | 451 | 385 | 808 | 8903 | 16241 | ND | *0.90* | 132 | 66.5 | 0.28 |
| 43 | Ceylon | NA | 1690 | *256* | *794* | 460 | 769 | 1064 | 9149 | 12640 | ND | 8.27 | 159 | 980 | 2.57 |
| 44 | Ceylon | Madagascar | *1217* | ND | *324* | 461 | 642 | 992 | 7654 | 7109 | *3.18* | *1.21* | 186 | 355 | 0.41 |
| 45 | Ceylon | Sri Lanka | 1503 | *142* | *414* | 558 | 302 | 924 | 10106 | 8962 | ND | 3.95 | 165 | 625 | 0.79 |
| 46 | Ceylon | India | *1275* | ND | *177* | 743 | *77.6* | 4354 | 6270 | 8667 | ND | *1.41* | 330 | 251 | 0.57 |
| 47 | Ceylon | NA | 1826 | ND | ND | 468 | 234 | *615* | 6418 | 12420 | ND | *1.08* | 126 | 20.1 | 0.22 |
| 48 | Ceylon | Sri Lanka | 1955 | ND | ND | 704 | 565 | 846 | 7106 | 14997 | ND | *0.62* | 86.2 | 30.0 | 0.30 |
| 49 | Ceylon | NA | 1775 | ND | *195* | 642 | 598 | 1162 | 7652 | 7320 | ND | *1.16* | 275 | 256 | 0.44 |
| 50 | Ceylon | Sri Lanka | 1732 | ND | *94.8* | 392 | 98.3 | 769 | 5999 | 14396 | ND | *1.10* | 213 | 105 | 0.35 |
| 51 | Ceylon | Sri Lanka | 1601 | *119* | *216* | 539 | 288 | 893 | 10141 | 8949 | *19.8* | 3.63 | 169 | 693 | 0.76 |
| 52 | Cassia | Indonesia | 2192 | ND | ND | 400 | *52.3* | 836 | 5879 | 13947 | ND | *1.21* | 129 | 31.0 | 0.23 |

b)

| **Sample id** | **Label variety** | **Origin** | **Cu LOQ**  **1.20**  **mg kg^-1^**  **U(k=2)**  **10.5 %** | **Zn LOQ**  **5.80**  **mg kg^-1^**  **U(k=2)**  **6.5 %** | **As LOQ 1.01 mg**  **kg^-1^**  **U(k=2)**  **15 %** | **Br LOQ 1.70 mg**  **kg^-1^**  **U(k=2)**  **22 %** | **Rb LOQ**  **4.20**  **mg kg^-1^**  **U(k=2)**  **5 %** | **Sr LOQ 1.19**  **mg kg^-1^**  **U(k=2)**  **8 %** | **Zr LOQ**  **10.1**  **mg kg^-1^**  **U(k=2)**  **5 %** | **Cd LOQ**  **0.70**  **mg kg^-1^**  **U(k=2)**  **25%** | **Ba LOQ**  **2.40**  **mg kg^-1^**  **U(k=2)**  **18 %** | **Pb LOQ**  **1.00**  **mg kg^-1^**  **U(k=2)**  **20 %** | **Hg LOQ**  **1.70**  **mg kg^-1^**  **U(k=2)**  **20.5 %** | **Se LOQ**  **0.37**  **mg kg^-1^**  **U(k=2)**  **9 %** | **Mo LOQ**  **0.41**  **mg kg^-1^**  **U(k=2)**  **15 %** |
| --- | --- | --- | --- | --- | --- | --- | --- | --- | --- | --- | --- | --- | --- | --- | --- |
| 1 | NA | NA | 3.42 | 20.4 | *0.81* | ND | 26.7 | 99.9 | ND | *0.15* | 44.2 | *0.09* | *0.34* | *0.19* | *0.10* |
| 2 | NA | NA | 3.00 | 17.0 | *0.76* | *0.46* | 19.4 | 79.6 | ND | *0.32* | 42.0 | ND | *0.11* | *0.20* | *0.01* |
| 3 | NA | NA | 3.04 | 16.6 | *0.77* | *0.26* | 22.9 | 84.3 | ND | *0.27* | 41.1 | ND | *0.17* | *0.13* | ND |
| 4 | NA | NA | 2.72 | 15.1 | *0.74* | ND | 19.5 | 99.5 | ND | *0.10* | 49.5 | *0.01* | *0.47* | *0.32* | ND |
| 5 | NA | NA | 3.89 | 10.3 | *0.77* | *0.52* | 34.3 | 51.2 | *0.28* | *0.42* | 154 | *0.07* | ND | *0.22* | *0.04* |
| 6 | NA | NA | 2.46 | 15.2 | *0.82* | *0.28* | 28.1 | 118 | ND | *0.03* | 39.1 | ND | ND | *0.24* | *0.06* |
| 7 | NA | NA | 3.06 | 13.9 | *0.76* | ND | 21.2 | 80.0 | ND | *0.56* | 37.4 | *0.08* | *0.36* | *0.21* | *0.03* |
| 8 | NA | NA | 2.61 | 14.4 | *0.81* | ND | 18.8 | 102 | ND | *0.03* | 43.8 | ND | *0.40* | *0.22* | *0.04* |
| 9 | NA | NA | 2.78 | 15.8 | *0.77* | 3.18 | 22.7 | 100 | ND | *0.09* | 48.2 | *0.11* | *0.55* | *0.12* | ND |
| 10 | NA | NA | 5.00 | 16.2 | *0.54* | 29.5 | 38.9 | 28.5 | *1.23* | *0.33* | 98.0 | 3.08 | *1.06* | *0.09* | *0.13* |
| 11 | Ceylon | Madagascar | 5.46 | 18.5 | *0.71* | *0.59* | 34.1 | 75.9 | *3.06* | *0.02* | 39.6 | *0.17* | *0.37* | *0.15* | *0.01* |
| 12 | Ceylon | Sri Lanka | 8.59 | 21.3 | *0.79* | 64.0 | 17.1 | 70.8 | *1.47* | *0.08* | 55.0 | *0.31* | *0.52* | *0.18* | *0.08* |
| 13 | NA | NA | 3.34 | 16.7 | *0.72* | ND | 17.7 | 80.1 | *0.89* | *0.16* | 54.3 | *0.10* | *0.38* | *0.19* | *0.14* |
| 14 | Ceylon | Madagascar | 5.23 | 15.0 | *0.73* | *0.09* | 34.1 | 129 | *4.23* | ND | 70.5 | ND | *0.38* | *0.21* | *0.09* |
| 15 | Burmanni | NA | 3.16 | 17.7 | *0.76* | ND | 22.1 | 89.5 | *1.10* | *0.01* | 48.3 | *0.02* | *0.34* | *0.15* | *0.06* |
| 16 | Aromaticum | NA | 2.68 | 15.9 | *0.77* | ND | 25.3 | 99.3 | ND | *0.19* | 45.2 | ND | *0.35* | *0.15* | ND |
| 17 | NA | Ceylan and  Indonesia | 5.16 | 18.7 | *0.77* | *0.35* | 29.3 | 68.8 | *4.94* | *0.02* | 59.0 | *0.12* | *0.35* | *0.26* | *0.15* |
| 18 | Ceylon | NA | 8.37 | 19.2 | *0.75* | 38.7 | 16.5 | 88.7 | *3.91* | *0.04* | 85.2 | *0.08* | *0.85* | *0.11* | *0.07* |
| 19 | Ceylon | NA | 4.06 | 9.55 | *0.74* | 21.0 | 32.6 | 43.9 | *0.39* | *0.19* | 87.1 | *0.82* | *0.78* | *0.09* | *0.19* |
| 20 | Ceylon | NA | 6.80 | 17.9 | *0.77* | *1.00* | 14.6 | 80.1 | *2.85* | *0.00* | 87.7 | *0.19* | *0.17* | *0.14* | *0.16* |
| 21 | Ceylon | NA | 6.82 | 18.5 | *0.73* | 2.25 | 33.6 | 75.8 | 13.0 | ND | 71.3 | *0.83* | *0.10* | *0.34* | *0.26* |
| 22 | Ceylon | NA | 5.84 | 19.3 | *0.72* | 14.3 | 24.1 | 97.7 | 24.4 | *0.37* | 85.7 | *0.51* | *0.69* | *0.12* | *0.20* |
| 23 | Cassia | NA | 3.03 | 13.9 | *0.78* | *1.33* | 14.6 | 81.5 | ND | ND | 29.8 | *0.07* | *0.37* | *0.19* | *0.11* |
| 24 | Cassia | Vietnam | 3.82 | 12.7 | *0.76* | ND | 24.4 | 63.1 | *0.55* | *0.48* | 78.7 | *0.17* | *0.29* | *0.19* | *0.18* |
| 25 | Ceylon | NA | 7.02 | 21.6 | *0.73* | *1.69* | 20.8 | 73.6 | 19.8 | *0.04* | 42.7 | *0.53* | *0.54* | *0.21* | *0.03* |
| 26 | NA | Vietnam | 7.29 | 18.7 | *0.62* | *0.78* | 32.7 | 42.9 | *5.22* | *0.32* | 116 | 2.30 | *0.32* | *0.15* | *0.10* |
| 27 | Burmanni | NA | 3.93 | 16.2 | *0.73* | ND | 27.1 | 86.5 | *0.39* | *0.11* | 56.2 | *0.06* | *0.30* | *0.25* | ND |
| 28 | Burmanni | NA | 2.92 | 13.5 | *0.74* | *0.26* | 24.1 | 83.0 | *0.38* | *0.33* | 35.3 | ND | *0.17* | *0.23* | *0.03* |
| 29 | Ceylon | NA | 9.55 | 25.6 | *0.70* | 3.83 | 34.5 | 64.3 | 19.7 | *0.40* | 57.8 | 1.17 | ND | *0.16* | *0.26* |
| 30 | Cassia | NA | 3.08 | 15.6 | *0.79* | *0.75* | 27.5 | 98.7 | ND | *0.31* | 43.3 | ND | ND | *0.23* | *0.04* |
| 31 | NA | Sri Lanka | 4.05 | 18.6 | *0.72* | *0.18* | 27.5 | 70.1 | *0.53* | *0.07* | 43.7 | *0.28* | *0.41* | *0.23* | ND |
| 32 | Ceylon | Sri Lanka | 8.67 | 11.7 | *0.76* | 1.95 | 19.6 | 53.0 | *0.36* | *0.13* | 52.9 | *0.33* | ND | *0.29* | *0.09* |
| 33 | Ceylon | Sri Lanka | 7.88 | 16.1 | *0.70* | 2.11 | 29.9 | 63.5 | *0.72* | *0.21* | 84.6 | 1.18 | ND | 0.84 | *0.21* |
| 34 | Ceylon | Sri Lanka | 11.1 | 20.7 | *0.75* | 2.07 | 27.2 | 73.3 | *0.25* | *0.24* | 50.3 | *0.16* | ND | *0.34* | *0.14* |
| 35 | Ceylon | Sri Lanka | 5.20 | 11.1 | *0.73* | 2.55 | 11.7 | 88.5 | *1.00* | *0.08* | 24.5 | ND | ND | 0.38 | *0.10* |
| 36 | Ceylon | Sri Lanka | 10.7 | 14.1 | *0.75* | 1.96 | 18.8 | 92.8 | *0.31* | *0.02* | 47.3 | *0.09* | ND | 0.62 | *0.10* |
| 37 | Ceylon | NA | 9.95 | 14.3 | *0.71* | 2.26 | 37.9 | 67.9 | *0.62* | *0.21* | 73.1 | *0.07* | ND | *0.35* | *0.16* |
| 38 |  | NA | 3.70 | 8.32 | *0.77* | 2.06 | 4.89 | 190 | ND | ND | 151 | *0.01* | *0.66* | 1.29 | *0.17* |
| 39 | Ceylon | Sri Lanka | 8.87 | 15.8 | *0.74* | 2.66 | 40.9 | 81.6 | *0.51* | *0.31* | 85.0 | *0.17* | ND | 0.46 | *0.20* |
| 40 | Ceylon | NA | 5.29 | 13.6 | *0.72* | 29.4 | 31.5 | 30.6 | *1.00* | 1.05 | 89.7 | 1.37 | ND | *0.03* | *0.12* |
| 41 | Ceylon | Sri Lanka | 7.85 | 15.2 | *0.70* | *0.27* | 66.6 | 81.4 | *2.16* | *0.17* | 58.3 | *0.42* | *0.33* | *0.17* | ND |
| 42 | Ceylon | Sri Lanka | 9.43 | 15.6 | *0.76* | 179 | 48.4 | 75.8 | *0.68* | *0.34* | 85.1 | *0.01* | *1.44* | 0.86 | *0.15* |
| 43 | Ceylon | NA | 11.6 | 25.0 | *0.71* | 2.67 | 32.8 | 87.3 | *9.64* | *0.05* | 89.9 | 1.10 | *0.38* | *0.22* | *0.14* |
| 44 | Ceylon | Madagascar | 6.44 | 17.6 | *0.77* | *1.02* | 38.7 | 75.8 | *2.55* | ND | 49.8 | *0.03* | *0.42* | *0.31* | *0.02* |
| 45 | Ceylon | Sri Lanka | 8.52 | 19.1 | *0.74* | *0.86* | 23.0 | 87.5 | *3.44* | *0.03* | 91.7 | *0.12* | *0.19* | *0.19* | *0.08* |
| 46 | Ceylon | India | 6.47 | 17.0 | *0.62* | *0.59* | 41.3 | 31.9 | *1.35* | *0.29* | 83.2 | 1.94 | *0.38* | *0.22* | *0.05* |
| 47 | Ceylon | NA | 8.49 | 11.3 | *0.69* | ND | 28.4 | 112 | ND | ND | 93.2 | *0.67* | *0.77* | 0.37 | ND |
| 48 | Ceylon | Sri Lanka | 7.48 | 14.3 | *0.74* | 2.51 | 11.3 | 72.5 | *0.35* | *0.36* | 44.3 | *0.48* | ND | *0.32* | *0.25* |
| 49 | Ceylon | NA | 7.54 | 16.8 | *0.73* | *1.35* | 39.9 | 81.6 | *2.56* | ND | 60.9 | *0.26* | *0.37* | *0.21* | *0.05* |
| 50 | Ceylon | Sri Lanka | 3.20 | 15.3 | *0.80* | *0.19* | 19.9 | 73.3 | *0.30* | *0.02* | 42.1 | *0.15* | *0.23* | *0.22* | *0.01* |
| 51 | Ceylon | Sri Lanka | 8.52 | 19.2 | *0.78* | *0.60* | 24.0 | 88.1 | *5.36* | *0.08* | 92.8 | *0.25* | *0.31* | *0.29* | *0.01* |
| 52 | Cassia | Indonesia | 2.88 | 17.3 | *0.79* | 12.1 | 21.7 | 99.2 | ND | *0.42* | 26.4 | *0.08* | *0.98* | *0.30* | *0.06* |

NA: Not available; ND: Not detected.

Numbers in grey are mass fractions below the LOQ of the method. Elements marked in orange are not significantly different and are not used for modelling purposes.
