# Supplementary material for: Use of elemental profiles determined by energy-dispersive X-ray fluorescence and multivariate analyses to detect adulteration in Ceylon cinnamon
Source: Anal Bioanal Chem. 2023 Aug 17;415(22):5437–49. doi: 10.1007/s00216-023-04817-1 (PMC10444698; doi:10.1007/s00216-023-04817-1)
Supplement: Supplementary file 2 — Supplementary file2 (DOCX 57 KB) [file 216_2023_4817_MOESM2_ESM.docx]

**Supplementary 2:** Relative intensities of volatile compounds found in Ceylon cinnamon and cassia samples.

a)

| **Sample id** | **Label variety** | **Origin** | **Bark/ground** | **Cinnamaldehyde** | **Eugenol** | **Benzyl benzoate** | **Coumarin** | **δ-cadinene** | **2-bornanone** | **γ-terpinene** | **α-pinene** | **Camphene** | **Benzaldehyde** | **β-pinene** | **6-methyl-5-heptene-2-one** | **Benzofuran** |
| --- | --- | --- | --- | --- | --- | --- | --- | --- | --- | --- | --- | --- | --- | --- | --- | --- |
| **1** | NA | NA | Ground | 3443 | ND | ND | 544 | 33.9 | ND | ND | 13.3 | 3.78 | 180 | 2.67 | ND | 2.93 |
| **2** | NA | NA | Ground | 2266 | 1.87 | ND | 408 | 17.7 | ND | ND | 3.56 | ND | 119 | ND | ND | ND |
| **3** | NA | NA | Ground | 3253 | ND | ND | 774 | 80.5 | ND | ND | 45.0 | 12.0 | 158 | 6.07 | ND | ND |
| **4** | NA | NA | Ground | 3580 | ND | ND | 625 | 232 | ND | ND | 3.77 | 2.03 | 148 | ND | ND | ND |
| **5** | NA | NA | Ground | 3462 | ND | ND | 780 | 1054 | ND | ND | 1.31 | ND | 321 | ND | 1.55 | 2.36 |
| **6** | NA | NA | Ground | 4168 | ND | ND | 620 | 575 | ND | ND | 3.33 | ND | 225 | ND | ND | ND |
| **7** | NA | NA | Ground | 2389 | ND | ND | 516 | 131 | 1.56 | ND | 33.7 | 8.06 | 151 | 4.28 | ND | 1.80 |
| **8** | NA | NA | Ground | 5575 | ND | ND | 978 | 1155 | 3.95 | 3.72 | 30.8 | 20.4 | 269 | 3.75 | ND | 3.08 |
| **9** | NA | NA | Ground | 3683 | ND | ND | 584 | 469 | ND | ND | 5.72 | ND | 151 | ND | ND | ND |
| **10** | NA | NA | Ground | 3554 | ND | ND | 116 | 791 | ND | ND | 1.49 | ND | 236 | ND | ND | ND |
| **11** | Ceylon | Madagascar | Ground | 3076 | 16.7 | 64.6 | 6.91 | ND | ND | ND | 2.40 | ND | 200 | ND | ND | ND |
| **12** | Ceylon | Sri Lanka | Ground | 2112 | 38.5 | 73.3 | ND | ND | ND | ND | 3.08 | ND | 145 | ND | ND | ND |
| **13** | NA | NON EU | Ground | 4245 | ND | ND | 935 | 295 | 7.12 | ND | 8.59 | 3.77 | 204 | ND | ND | ND |
| **14** | Ceylon | Madagascar | Ground | 2751 | 91.1 | 165 | 11.1 | ND | 3.27 | ND | 3.03 | ND | 122 | ND | ND | ND |
| **15** | Burmanni | NA | Ground | 3217 | ND | ND | 583 | 46.1 | 3.15 | ND | 3.70 | ND | 106 | ND | ND | ND |
| **16** | Aromaticum | NA | Ground | 3338 | ND | ND | 509 | 283 | ND | ND | 5.42 | 3.22 | 176 | ND | ND | 3.94 |
| **17** | NA | Ceylan and Indonesia | Ground | 2713 | 5.16 | 12.9 | 417 | 117 | 36.4 | ND | 6.02 | 1.61 | 195 | 2.74 | ND | 2.75 |
| **18** | Ceylon | NON EU | Ground | 3300 | 23.7 | 7.32 | 9.03 | ND | ND | ND | 6.14 | ND | 166 | ND | ND | 4.26 |
| **19** | Ceylon | NON EU | Ground | 2233 | **ND** | **ND** | **203** | **391** | ND | ND | ND | ND | 144 | ND | 6.10 | ND |
| **20** | Ceylon | NON EU | Ground | 2869 | 15.6 | 5.48 | 5.12 | ND | ND | ND | 5.44 | ND | 135 | ND | ND | 3.01 |
| **21** | Ceylon | NA | Ground | 2566 | 24.2 | 54.6 | 261 | 11.1 | 23.9 | ND | 8.78 | 2.40 | 66.6 | 3.08 | ND | ND |
| **22** | Ceylon | NA | Ground | 2536 | 34.9 | 17.1 | 4.07 | ND | 270 | ND | 3.52 | ND | 51.5 | ND | ND | ND |
| **23** | Cassia | NA | Ground | 2091 | ND | ND | 381 | 220 | ND | ND | 8.02 | 1.94 | 88.8 | 2.01 | ND | 1.84 |
| **24** | Cassia | Vietnam | Ground | 2316 | 11.9 | ND | 453 | 972 | ND | ND | 1.37 | ND | 111 | ND | 1.56 | 1.48 |
| **25** | Ceylon | NA | Ground | 2501 | 27.4 | 92.1 | 8.35 | 20.2 | 121 | 3.78 | 27.9 | 7.52 | 183 | 4.29 | ND | 2.48 |
| **26** | NA | Vietnam | Ground | 1754 | ND | ND | 484 | 36.1 | ND | ND | ND | ND | 62.1 | ND | ND | 1.54 |
| **27** | Burmanni | ND | Ground | 3338 | ND | ND | 510 | 86.9 | ND | ND | 6.51 | 2.20 | 131 | ND | ND | ND |
| **28** | Burmanni | ND | Ground | 3007 | ND | ND | 685 | 262 | ND | ND | 14.1 | 4.26 | 171 | ND | ND | ND |
| **29** | Ceylon | NA | Ground | 2410 | 33.2 | 45.9 | 11.5 | 9.22 | 102 | 2.61 | 32.0 | 8.29 | 154 | 5.29 | ND | ND |
| **30** | Cassia | NA | Ground | 3516 | ND | ND | 687 | 133 | ND | ND | 6.31 | ND | 165 | ND | ND | 2.33 |
| **31** | NA | Sri Lanka | Ground | 2972 | ND | ND | 619 | 365 | ND | ND | 23.1 | 6.36 | 140 | 5.17 | ND | 2.22 |
| **32** | Ceylon | Sri Lanka | Bark | 2607 | 168 | 211 | ND | 15.9 | ND | 3.02 | 28.4 | 5.39 | 166 | 3.78 | ND | ND |
| **33** | Ceylon | Sri Lanka | Bark | 2695 | 98.0 | 77.3 | 10.5 | 12.3 | ND | 3.77 | 15.0 | 3.77 | 154 | ND | ND | ND |
| **34** | Ceylon | Sri Lanka | Bark | 2851 | 44.4 | 81.5 | 9.32 | 14.7 | ND | 5.28 | 26.1 | 6.96 | 264 | 2.70 | ND | ND |
| **35** | Ceylon | Sri Lanka | Bark | 2101 | 567 | 177 | 8.46 | 4.84 | ND | 4.53 | 25.5 | 4.93 | 172 | 2.66 | ND | ND |
| **36** | Ceylon | Sri Lanka | Bark | 2452 | 101 | 67.5 | 4.97 | 7.12 | ND | 2.60 | 12.7 | 2.76 | 132 | 3.01 | ND | ND |
| **37** | Ceylon | NA | Bark | 2883 | 72.9 | 200 | 5.00 | 7.04 | ND | 4.10 | 31.8 | 6.71 | 254 | 4.94 | ND | ND |
| **38** | NA | NA | Bark | 3565 | 18.4 | 33.2 | 20.1 | 36.9 | ND | 18.4 | 14.8 | 3.62 | 223 | 8.55 | ND | ND |
| **39** | Ceylon | Sri Lanka | Bark | 5442 | 1288 | 171 | 2.73 | 23.4 | ND | 2.80 | ND | ND | 128 | ND | ND | ND |
| **40** | Ceylon | NA | Ground | 2233 | **ND** | **ND** | **378** | **807** | ND | ND | ND | ND | 84.7 | ND | 4.86 | ND |
| **41** | Ceylon | Sri Lanka | Ground | 2765 | 98.0 | 104 | 2.11 | 7.48 | ND | ND | 3.59 | ND | 223 | ND | ND | ND |
| **42** | Ceylon | Sri Lanka | Bark | 3964 | 348 | 195 | ND | 28.8 | ND | 3.93 | 6.00 | ND | 162 | ND | ND | ND |
| **43** | Ceylon | NA | Ground | 2870 | 39.1 | 25.7 | 6.38 | 15.5 | ND | ND | 4.50 | ND | 282 | ND | ND | 8.22 |
| **44** | Ceylon | Madagascar | Ground | 1842 | 77.4 | 48.8 | 4.20 | 4.75 | 9.28 | ND | 4.25 | ND | 112 | ND | ND | ND |
| **45** | Ceylon | Sri Lanka | Ground | 2753 | 34.4 | 8.86 | 4.11 | 27.2 | 6.61 | 2.16 | 8.20 | 3.10 | 182 | 3.11 | ND | 3.57 |
| **46** | Ceylon | India | Ground | 2390 | **ND** | **2.73** | **541** | **691** | ND | ND | ND | ND | 130 | ND | 5.46 | 1.10 |
| **47** | Ceylon | NA | Bark | 3930 | 305 | 221 | 7.13 | 6.26 | ND | 6.17 | 59.8 | 12.2 | 216 | 6.90 | ND | ND |
| **48** | Ceylon | Sri Lanka | Bark | 2164 | 435 | 22.2 | 12.3 | 20.5 | ND | 16.4 | 74.1 | 14.1 | 318 | 11.4 | ND | ND |
| **49** | Ceylon | NA | Ground | 2233 | 41.7 | 31.7 | 5.15 | 2.62 | 2.17 | ND | 4.03 | ND | 81.7 | ND | ND | ND |
| **50** | Ceylon | Sri Lanka | Ground | 3220 | **ND** | **ND** | **765** | **352** | ND | ND | 4.50 | ND | 191 | ND | ND | ND |
| **51** | Ceylon | Sri Lanka | Ground | 2692 | 31.7 | 7.80 | 13.1 | 28.0 | 6.27 | 2.63 | 10.7 | 3.88 | 198 | 4.27 | ND | 4.56 |
| **52** | Cassia | Indonesia | Bark | 3355 | ND | ND | 735 | 278 | ND | 6.15 | 31.7 | 6.93 | 87.4 | 15.6 | ND | ND |

b)

| **Sample id** | **Label variety** | **Origin** | **Bark/ground** | **α-phellandrene** | **o-cymene** | **p-cymene** | **D-limonene** | **Eucalyptol** | **β-phellandrene** | **Benzyl alcohol** | **Benzeneacetaldehyde** | **Benzene, (methoxymethyl)-** | **Acetophenone** | **Linaool oxide** | **p-cymenene** | **Benzoic acid, methyl ester** |
| --- | --- | --- | --- | --- | --- | --- | --- | --- | --- | --- | --- | --- | --- | --- | --- | --- |
| **1** | NA | NA | Ground | ND | 5.20 | ND | 7.14 | 67.4 | ND | ND | ND | 11.0 | 6.91 | ND | 2.72 | ND |
| **2** | NA | NA | Ground | ND | 2.87 | ND | 1.84 | 37.0 | ND | ND | ND | 9.20 | 10.2 | ND | ND | 2.60 |
| **3** | NA | NA | Ground | ND | 7.19 | 72.9 | 16.4 | 134 | ND | ND | ND | 9.11 | 6.48 | ND | ND | 2.40 |
| **4** | NA | NA | Ground | ND | 3.94 | ND | 2.88 | 66.3 | ND | ND | 15.1 | ND | 9.68 | ND | ND | 3.69 |
| **5** | NA | NA | Ground | ND | ND | ND | ND | 9.64 | ND | ND | ND | 24.8 | 22.8 | ND | ND | 4.57 |
| **6** | NA | NA | Ground | ND | 7.46 | ND | 6.67 | 119 | ND | ND | 17.8 | ND | 10.9 | ND | 6.20 | 6.89 |
| **7** | NA | NA | Ground | ND | 5.92 | ND | 10.7 | 163 | ND | ND | 11.7 | ND | 73.6 | 3.65 | ND | 5.58 |
| **8** | NA | NA | Ground | ND | 15.0 | ND | 21.3 | 192 | ND | ND | 16.4 | ND | 23.6 | ND | 6.73 | 7.67 |
| **9** | NA | NA | Ground | ND | 4.54 | ND | 3.34 | 49.5 | ND | ND | ND | 12.2 | 7.67 | ND | ND | 3.38 |
| **10** | NA | NA | Ground | ND | 1.83 | 8.87 | 82.3 | 7.56 | ND | ND | 8.16 | ND | 11.4 | ND | ND | 3.30 |
| **11** | Ceylon | Madagascar | Ground | ND | 14.3 | 28.2 | 3.06 | ND | ND | 11.9 | ND | 6.01 | 14.4 | ND | ND | 10.66 |
| **12** | Ceylon | Sri Lanka | Ground | 7.75 | 39.9 | 103 | 13.0 | 13.9 | 6.50 | 6.46 | ND | 5.20 | 7.16 | 3.03 | 11.0 | 2.07 |
| **13** | NA | NON EU | Ground | ND | 6.41 | ND | 7.53 | 128 | ND | ND | 14.7 | ND | 13.2 | ND | ND | 4.02 |
| **14** | Ceylon | Madagascar | Ground | 2.36 | 24.5 | 65.7 | 4.29 | 3.07 | 6.01 | 12.4 | ND | 5.16 | 10.5 | 3.33 | 8.52 | 6.44 |
| **15** | Burmanni | NA | Ground | ND | 5.37 | ND | 92.9 | 47.5 | ND | ND | 12.9 | ND | 11.3 | 8.49 | ND | 3.71 |
| **16** | Aromaticum | NA | Ground | ND | 7.37 | ND | 7.66 | 128 | ND | ND | 13.6 | ND | 7.12 | ND | 4.07 | 4.94 |
| **17** | NA | Ceylan and Indonesia | Ground | ND | 8.37 | 33.0 | 3.51 | 27.1 | 1.63 | ND | ND | 11.0 | 10.6 | 1.73 | 3.19 | 2.52 |
| **18** | Ceylon | NON EU | Ground | 2.64 | 32.2 | 92.6 | 11.8 | 17.2 | 11.7 | 5.62 | ND | 4.88 | 6.87 | ND | 6.73 | 7.33 |
| **19** | Ceylon | NON EU | Ground | ND | ND | 1.12 | ND | ND | ND | ND | 9.12 | ND | 8.38 | ND | ND | 2.26 |
| **20** | Ceylon | NON EU | Ground | 3.12 | 18.2 | 48.5 | 7.93 | 3.12 | 10.1 | ND | ND | 4.24 | 8.28 | 4.41 | 6.24 | 6.64 |
| **21** | Ceylon | NA | Ground | 3.43 | 23.8 | 63.8 | 7.32 | 12.5 | 6.63 | 1.90 | ND | 4.54 | 5.83 | 3.20 | 5.98 | 2.01 |
| **22** | Ceylon | NA | Ground | 2.12 | 16.8 | 51.1 | 2.99 | 18.1 | 3.02 | 12.6 | ND | 2.41 | 3.50 | ND | 5.01 | ND |
| **23** | Cassia | NA | Ground | ND | 3.36 | ND | 4.68 | 59.6 | ND | ND | ND | 6.28 | 4.62 | 1.79 | 1.91 | 2.15 |
| **24** | Cassia | Vietnam | Ground | ND | ND | ND | 2.39 | 10.6 | ND | ND | 10.0 | ND | 7.10 | ND | ND | 2.90 |
| **25** | Ceylon | NA | Ground | 7.52 | 78.7 | 222 | 33.4 | 50.5 | 18.9 | ND | 4.09 | ND | 10.4 | 8.20 | 12.2 | 2.41 |
| **26** | NA | Vietnam | Ground | ND | ND | ND | ND | ND | ND | ND | 7.32 | ND | 4.15 | ND | ND | 2.46 |
| **27** | Burmanni | ND | Ground | ND | 2.31 | ND | ND | 33.0 | ND | ND | 10.2 | ND | 7.54 | ND | ND | 2.74 |
| **28** | Burmanni | ND | Ground | ND | 4.69 | ND | 8.08 | 81.6 | ND | ND | 11.3 | ND | 8.44 | ND | ND | 4.60 |
| **29** | Ceylon | NA | Ground | 6.72 | 45.5 | 135 | 22.1 | 32.0 | 18.7 | 8.28 | ND | 4.48 | 8.28 | 4.09 | 7.52 | 3.00 |
| **30** | Cassia | NA | Ground | ND | 4.64 | ND | 4.03 | 79.6 | ND | ND | 12.0 | ND | 6.94 | ND | ND | 4.04 |
| **31** | NA | Sri Lanka | Ground | ND | 3.64 | ND | 6.06 | 68.9 | ND | ND | ND | 8.94 | 7.32 | ND | ND | 2.55 |
| **32** | Ceylon | Sri Lanka | Bark | 7.83 | 50.7 | 167 | 19.3 | 32.8 | 43.7 | ND | 5.12 | ND | 7.67 | 8.12 | 6.44 | 2.73 |
| **33** | Ceylon | Sri Lanka | Bark | 9.89 | 49.6 | 128 | 17.8 | 18.6 | 23.2 | 2.97 | 5.78 | ND | 7.82 | 5.72 | 15.5 | 5.13 |
| **34** | Ceylon | Sri Lanka | Bark | 9.34 | 95.8 | 245 | 26.0 | 25.4 | 22.2 | ND | 5.02 | ND | 14.8 | 10.0 | 15.8 | 5.61 |
| **35** | Ceylon | Sri Lanka | Bark | 12.6 | 98.8 | 261 | 34.0 | 16.8 | 66.1 | 3.18 | 7.18 | ND | 7.84 | 6.66 | 14.0 | 10.20 |
| **36** | Ceylon | Sri Lanka | Bark | 7.06 | 37.4 | 111 | 16.1 | 12.3 | 41.5 | 2.66 | 5.55 | ND | 7.98 | 3.67 | 7.71 | 4.68 |
| **37** | Ceylon | NA | Bark | 9.42 | 60.1 | 175 | 22.4 | 36.0 | 39.9 | ND | 8.53 | ND | 10.2 | 25.8 | 12.5 | 10.42 |
| **38** | NA | NA | Bark | 19.7 | 109.36 | 263 | ND | 12.1 | 141 | ND | 4.05 | ND | 8.19 | 6.71 | 20.7 | ND |
| **39** | Ceylon | Sri Lanka | Bark | 16.60 | 40.6 | 156 | ND | 10.7 | 109 | 9.16 | ND | 6.48 | 3.24 | ND | 12.3 | ND |
| **40** | Ceylon | NA | Ground | ND | ND | 2.07 | 1.59 | ND | ND | ND | 6.17 | ND | 5.49 | ND | ND | 1.62 |
| **41** | Ceylon | Sri Lanka | Ground | 2.43 | 11.4 | 29.5 | 3.48 | 2.75 | 4.91 | 3.01 | 4.59 | ND | 9.74 | 2.71 | ND | 10.32 |
| **42** | Ceylon | Sri Lanka | Bark | 12.8 | 40.2 | 96.2 | 13.0 | 11.4 | 21.7 | 5.97 | 7.10 | ND | 5.19 | 4.05 | 19.7 | 17.91 |
| **43** | Ceylon | NA | Ground | 4.04 | 30.2 | 75.6 | 7.85 | 5.38 | 6.33 | 57.2 | ND | 6.02 | 6.01 | 3.25 | ND | 12.87 |
| **44** | Ceylon | Madagascar | Ground | 3.01 | 11.0 | 27.0 | 2.99 | 3.86 | 5.08 | 16.9 | ND | 3.94 | 6.88 | 1.80 | ND | 15.59 |
| **45** | Ceylon | Sri Lanka | Ground | 4.10 | 65.0 | 176 | 42.3 | 6.94 | 32.8 | ND | 4.10 | ND | 7.67 | 8.41 | ND | 28.45 |
| **46** | Ceylon | India | Ground | ND | 2.88 | 9.59 | ND | 6.74 | ND | ND | ND | 14.0 | 15.8 | ND | ND | 5.61 |
| **47** | Ceylon | NA | Bark | 11.2 | 82.0 | 229 | 33.9 | 23.0 | 90.0 | ND | 9.50 | ND | 9.13 | 6.87 | 9.88 | 7.66 |
| **48** | Ceylon | Sri Lanka | Bark | 36.3 | 136.86 | 393 | 60.8 | 35.2 | 221 | ND | 6.47 | ND | 4.43 | 4.76 | ND | 16.91 |
| **49** | Ceylon | NA | Ground | 3.59 | 16.2 | 41.5 | 4.72 | 6.76 | 5.73 | 10.9 | ND | 3.48 | 5.58 | 2.68 | ND | 8.02 |
| **50** | Ceylon | Sri Lanka | Ground | ND | 3.39 | ND | ND | 31.4 | ND | ND | 12.4 | ND | 173 | ND | ND | 4.04 |
| **51** | Ceylon | Sri Lanka | Ground | 4.32 | 80.2 | 215 | 52.7 | 9.52 | 36.4 | 2.28 | ND | 4.13 | 4.61 | 11.6 | ND | 22.32 |
| **52** | Cassia | Indonesia | Bark | ND | 14.5 | ND | 65.9 | 315 | ND | ND | ND | 9.89 | 11.9 | ND | 4.50 | 1.96 |

c)

| **Sample id** | **Label variety** | **Origin** | **Bark/ground** | **β-linalool** | **Benzofuran, 2-methyl-** | **Hydrocinnamaldehyde** | **Borneol** | **Terpinen-4-ol** | **α-terpineol** | **Sabinol** | **3-phenylpropanol** | **Cumic aldehyde** | **Carvone** | **Bornyl acetate** | **Anethole** | **Safrole** |
| --- | --- | --- | --- | --- | --- | --- | --- | --- | --- | --- | --- | --- | --- | --- | --- | --- |
| **1** | NA | NA | Ground | 3.5 | 9.31 | 90.8 | 14.8 | 38.7 | 56.0 | ND | 3.00 | ND | ND | 131 | ND | ND |
| **2** | NA | NA | Ground | 2.6 | 4.80 | 87.7 | ND | 37.0 | 48.8 | ND | 5.31 | ND | ND | 55.5 | ND | ND |
| **3** | NA | NA | Ground | 3.3 | 2.30 | 73.6 | ND | 33.6 | 44.4 | ND | 3.64 | ND | ND | 132 | ND | ND |
| **4** | NA | NA | Ground | 2.5 | 6.76 | 152 | ND | 44.3 | 59.6 | ND | 2.99 | ND | ND | 77.9 | ND | ND |
| **5** | NA | NA | Ground | ND | 21.01 | 311 | 2.60 | 4.65 | 12.0 | ND | 16.4 | ND | ND | ND | ND | ND |
| **6** | NA | NA | Ground | 4.4 | 15.74 | 296 | 6.40 | 92.2 | 80.7 | ND | 4.34 | ND | ND | 262 | ND | ND |
| **7** | NA | NA | Ground | 9.3 | 5.82 | 108 | ND | 31.7 | 64.7 | ND | 86.1 | ND | ND | 71.4 | ND | ND |
| **8** | NA | NA | Ground | 3.4 | 4.79 | 104 | ND | 59.9 | 63.1 | ND | 28.5 | ND | ND | 209 | ND | ND |
| **9** | NA | NA | Ground | 2.9 | 10.25 | 123 | ND | 56.4 | 63.3 | ND | 11.6 | ND | ND | 96.1 | ND | ND |
| **10** | NA | NA | Ground | ND | 1.40 | 100 | ND | ND | 3.07 | ND | ND | ND | 87.0 | ND | ND | ND |
| **11** | Ceylon | Madagascar | Ground | 11.8 | 0.00 | 28.5 | ND | 11.5 | 18.0 | ND | 7.51 | ND | ND | ND | 5.22 | ND |
| **12** | Ceylon | Sri Lanka | Ground | 10.0 | 0.00 | 33.6 | ND | 16.4 | 40.1 | ND | 1.85 | 5.70 | 8.97 | ND | ND | ND |
| **13** | NA | NON EU | Ground | ND | 0.00 | 99.5 | ND | 37.9 | 54.2 | ND | 106 | ND | ND | 81.9 | ND | ND |
| **14** | Ceylon | Madagascar | Ground | 50.2 | 0.00 | 59.5 | ND | 19.7 | 34.8 | ND | 5.15 | 13.0 | 2.60 | ND | ND | ND |
| **15** | Burmanni | NA | Ground | 64.7 | 7.52 | 119 | ND | 28.8 | 48.2 | ND | 139 | ND | ND | 36.3 | ND | ND |
| **16** | Aromaticum | NA | Ground | 3.4 | 12.58 | 152 | 6.39 | 49.7 | 56.9 | ND | ND | ND | ND | 145 | ND | ND |
| **17** | NA | Ceylan and Indonesia | Ground | 10.0 | 4.38 | 93.1 | ND | 19.3 | 26.7 | ND | 11.2 | ND | ND | 44.0 | ND | ND |
| **18** | Ceylon | NON EU | Ground | 22.1 | 0.00 | 23.2 | ND | 6.46 | 17.2 | ND | 2.95 | ND | ND | ND | ND | ND |
| **19** | Ceylon | NON EU | Ground | ND | 16.48 | 268 | ND | ND | ND | ND | 4.20 | ND | ND | ND | ND | ND |
| **20** | Ceylon | NON EU | Ground | 40.4 | 0.00 | 18.6 | ND | 12.0 | 21.4 | ND | 4.33 | ND | ND | ND | ND | ND |
| **21** | Ceylon | NA | Ground | 22.2 | 3.46 | 44.3 | ND | 15.4 | 20.2 | ND | 2.88 | ND | ND | ND | ND | ND |
| **22** | Ceylon | NA | Ground | 7.6 | 0.00 | 19.3 | 2.11 | 7.65 | 12.5 | ND | 1.95 | ND | ND | ND | 9.01 | 6.90 |
| **23** | Cassia | NA | Ground | 9.8 | 2.90 | 38.9 | 4.69 | 28.6 | 47.6 | ND | 4.55 | 3.70 | ND | 40.0 | ND | ND |
| **24** | Cassia | Vietnam | Ground | ND | 10.77 | 121 | ND | ND | 2.56 | ND | 2.44 | ND | ND | ND | ND | ND |
| **25** | Ceylon | NA | Ground | 95.2 | 0.00 | 21.4 | ND | 39.3 | 55.4 | ND | 5.10 | 18.3 | ND | ND | 6.08 | 3.73 |
| **26** | NA | Vietnam | Ground | ND | 5.45 | 57.8 | ND | ND | ND | ND | 3.59 | ND | ND | ND | ND | ND |
| **27** | Burmanni | ND | Ground | ND | 6.47 | 92.7 | ND | 18.7 | 27.0 | ND | 17.7 | ND | ND | 46.7 | ND | ND |
| **28** | Burmanni | ND | Ground | ND | 2.48 | 91.6 | ND | 23.8 | 33.3 | ND | 26.0 | ND | ND | 71.1 | ND | ND |
| **29** | Ceylon | NA | Ground | 49.5 | 0.00 | 20.0 | 7.96 | 33.9 | 39.3 | ND | 4.62 | ND | ND | ND | 4.25 | 5.19 |
| **30** | Cassia | NA | Ground | 3.9 | 13.93 | 139 | 20.0 | 46.9 | 59.3 | ND | 5.66 | ND | ND | 129 | ND | ND |
| **31** | NA | Sri Lanka | Ground | 6.5 | 5.31 | 67.1 | 15.7 | 31.3 | 46.1 | ND | 9.03 | ND | ND | 142 | ND | ND |
| **32** | Ceylon | Sri Lanka | Bark | 247 | 0.00 | 88.0 | ND | 50.6 | 64.6 | ND | 7.72 | ND | ND | ND | ND | ND |
| **33** | Ceylon | Sri Lanka | Bark | 36.7 | 3.51 | 93.1 | ND | 38.6 | 74.4 | 5.31 | 4.29 | 3.71 | ND | ND | ND | ND |
| **34** | Ceylon | Sri Lanka | Bark | 45.6 | 0.00 | 45.1 | ND | 50.0 | 52.6 | ND | 3.75 | 9.09 | ND | ND | ND | ND |
| **35** | Ceylon | Sri Lanka | Bark | 160 | 2.62 | 116 | ND | 58.4 | 67.4 | 4.60 | 7.01 | ND | ND | ND | ND | 5.75 |
| **36** | Ceylon | Sri Lanka | Bark | 125 | 2.23 | 104 | ND | 42.2 | 57.6 | ND | 5.31 | ND | ND | ND | ND | ND |
| **37** | Ceylon | NA | Bark | 380 | 2.05 | 112 | ND | 67.8 | 71.6 | 2.27 | 8.23 | ND | 23.4 | ND | 7.58 | ND |
| **38** | NA | NA | Bark | 308 | 0.00 | 65.2 | ND | 106 | 40.0 | ND | ND | 11.4 | ND | ND | ND | 18.7 |
| **39** | Ceylon | Sri Lanka | Bark | 368 | 6.11 | 156 | 5.50 | 172 | 161 | 13.2 | 5.94 | ND | ND | ND | ND | ND |
| **40** | Ceylon | NA | Ground | ND | 15.68 | 122 | ND | ND | ND | ND | 3.59 | ND | ND | ND | ND | ND |
| **41** | Ceylon | Sri Lanka | Ground | 62.8 | 0.00 | 90.0 | ND | 19.0 | 35.9 | ND | 5.58 | ND | ND | ND | ND | ND |
| **42** | Ceylon | Sri Lanka | Bark | 530 | 7.12 | 131 | 5.06 | 56.8 | 103 | 6.36 | 4.88 | ND | 3.31 | ND | ND | ND |
| **43** | Ceylon | NA | Ground | 24.3 | 0.00 | 22.6 | ND | 5.56 | 22.6 | ND | ND | ND | ND | ND | ND | ND |
| **44** | Ceylon | Madagascar | Ground | 25.4 | 0.00 | 39.8 | ND | 10.5 | 24.5 | ND | 11.9 | ND | ND | ND | ND | ND |
| **45** | Ceylon | Sri Lanka | Ground | 246 | 0.00 | 25.1 | 7.40 | 65.7 | 34.0 | ND | 6.50 | ND | ND | ND | ND | ND |
| **46** | Ceylon | India | Ground | ND | 76.09 | 376 | ND | 7.99 | 3.86 | ND | 19.8 | ND | ND | ND | 64.4 | ND |
| **47** | Ceylon | NA | Bark | 190 | 3.38 | 128 | ND | 58.4 | 60.0 | ND | 14.7 | ND | ND | ND | ND | ND |
| **48** | Ceylon | Sri Lanka | Bark | 332 | 5.85 | 188 | 3.78 | 112 | 93.6 | 2.40 | 7.10 | ND | ND | 2.40 | ND | ND |
| **49** | Ceylon | NA | Ground | 33.9 | 0.00 | 23.8 | 1.85 | 11.4 | 22.9 | ND | 5.40 | ND | ND | ND | ND | ND |
| **50** | Ceylon | Sri Lanka | Ground | ND | 0.00 | 103 | ND | 14.9 | 28.1 | ND | 7.53 | ND | ND | 62.3 | ND | ND |
| **51** | Ceylon | Sri Lanka | Ground | 264 | 0.00 | 26.0 | 7.87 | 69.4 | 36.5 | ND | 6.09 | ND | ND | ND | ND | ND |
| **52** | Cassia | Indonesia | Bark | 16.4 | 44.02 | 221 | 25.6 | 132 | 116 | ND | 66.9 | ND | ND | 232 | ND | ND |

d)

| **Sample id** | **Label variety** | **Origin** | **Bark/ground** | **Thymol** | **Cinnamyl alcohol** | **Ylangene** | **β-guaiene** | **Cyclosativene** | **α-copaene** | **β-elemene** | **Sativene** | **Methyleugenol** | **α-bergamotene** | **Isosativene** | **Caryophyllene** | **Cinnamyl acetate** |
| --- | --- | --- | --- | --- | --- | --- | --- | --- | --- | --- | --- | --- | --- | --- | --- | --- |
| **1** | NA | NA | Ground | ND | ND | ND | ND | 45.5 | 1061 | 27.1 | 20.9 | ND | 27.3 | ND | 86.9 | 203 |
| **2** | NA | NA | Ground | ND | ND | ND | 8.73 | 14.0 | 262 | 7.36 | ND | ND | 43.4 | ND | 11.9 | ND |
| **3** | NA | NA | Ground | ND | ND | ND | 86.3 | 46.0 | 869 | 16.8 | 16.5 | ND | 80.5 | ND | 117 | 166 |
| **4** | NA | NA | Ground | ND | ND | ND | 10.6 | 8.73 | 142 | 4.03 | 5.94 | ND | 37.4 | ND | 40.3 | ND |
| **5** | NA | NA | Ground | ND | ND | 56.2 | 15.2 | 504 | 1441 | 352 | 169 | ND | ND | 178 | 138 | 21.8 |
| **6** | NA | NA | Ground | ND | ND | ND | 8.07 | 77.5 | 1271 | 33.8 | 23.5 | ND | 234 | ND | 480 | ND |
| **7** | NA | NA | Ground | ND | ND | ND | 9.59 | 11.1 | 239 | 6.52 | 2.64 | ND | 22.7 | ND | 48.5 | 3.84 |
| **8** | NA | NA | Ground | ND | ND | ND | 19.4 | 91.4 | 1607 | 39.9 | 52.6 | ND | 270 | ND | 494 | ND |
| **9** | NA | NA | Ground | ND | ND | ND | 9.48 | 32.3 | 643 | 18.3 | 17.9 | ND | 108 | 6.22 | 223 | ND |
| **10** | NA | NA | Ground | ND | ND | 44.6 | ND | 431 | 1654 | 130 | 281 | ND | 118 | 128 | 78.3 | ND |
| **11** | Ceylon | Madagascar | Ground | ND | ND | ND | ND | ND | 17.2 | ND | ND | 5.24 | ND | ND | 45.8 | 7.38 |
| **12** | Ceylon | Sri Lanka | Ground | 9.63 | ND | ND | ND | ND | 114 | 3.00 | ND | ND | ND | ND | 103 | 24.6 |
| **13** | NA | NON EU | Ground | ND | ND | ND | 26.8 | 19.8 | 282 | 5.95 | 11.3 | ND | 81.0 | ND | 43.5 | ND |
| **14** | Ceylon | Madagascar | Ground | ND | ND | ND | ND | ND | 6.45 | ND | ND | 7.77 | ND | ND | 61.5 | 79.5 |
| **15** | Burmanni | NA | Ground | ND | ND | ND | 14.4 | 8.15 | 124 | ND | ND | ND | 6.71 | ND | 13.0 | ND |
| **16** | Aromaticum | NA | Ground | ND | ND | ND | 10.6 | 79.2 | 1109 | 43.6 | 31.0 | ND | 130 | ND | 390 | ND |
| **17** | NA | Ceylan and Indonesia | Ground | ND | ND | ND | 16.9 | 83.2 | 1024 | 48.1 | 64.6 | ND | ND | ND | 111 | 11.4 |
| **18** | Ceylon | NON EU | Ground | ND | ND | ND | ND | ND | 44.3 | ND | ND | ND | ND | ND | 121 | 2.60 |
| **19** | Ceylon | NON EU | Ground | ND | ND | ND | 52.9 | 169 | 844 | 74.5 | 185 | ND | ND | 57.8 | 38.0 | 22.7 |
| **20** | Ceylon | NON EU | Ground | ND | ND | ND | ND | ND | 18.9 | ND | ND | ND | ND | ND | 97.3 | 8.73 |
| **21** | Ceylon | NA | Ground | 2.73 | ND | ND | 2.28 | 5.92 | 32.5 | ND | 10.2 | ND | ND | ND | 15.4 | 14.2 |
| **22** | Ceylon | NA | Ground | ND | ND | ND | ND | ND | 6.24 | ND | ND | 7.58 | ND | ND | 25.8 | 5.10 |
| **23** | Cassia | NA | Ground | ND | ND | ND | 169 | 45.2 | 310 | 14.3 | 7.10 | ND | 41.6 | ND | 50.2 | ND |
| **24** | Cassia | Vietnam | Ground | ND | ND | ND | ND | 150 | 907 | 104 | 152 | ND | ND | 66.4 | 237 | ND |
| **25** | Ceylon | NA | Ground | 7.83 | ND | ND | ND | ND | 156 | 2.42 | ND | ND | ND | ND | 520 | 62.3 |
| **26** | NA | Vietnam | Ground | ND | ND | ND | ND | 8.98 | 71.8 | 1.50 | 10.6 | ND | ND | ND | 3.54 | 3.65 |
| **27** | Burmanni | ND | Ground | ND | ND | ND | ND | 42.6 | 688 | 20.0 | 42.0 | ND | ND | ND | 38.3 | 24.9 |
| **28** | Burmanni | ND | Ground | 5.23 | ND | ND | ND | 23.1 | 481 | 13.6 | 11.4 | ND | 40.9 | ND | 102 | ND |
| **29** | Ceylon | NA | Ground | ND | 8.59 | ND | ND | 6.93 | 240 | 3.54 | ND | ND | ND | ND | 353 | 54.8 |
| **30** | Cassia | NA | Ground | ND | ND | ND | 22.8 | 38.9 | 805 | 21.9 | 19.9 | ND | 48.1 | ND | 233 | ND |
| **31** | NA | Sri Lanka | Ground | ND | ND | ND | 29.7 | 85.3 | 1147 | 27.6 | 40.9 | ND | 130 | ND | 234 | 15.5 |
| **32** | Ceylon | Sri Lanka | Bark | ND | 53.5 | ND | ND | ND | 127 | ND | ND | ND | ND | ND | 862 | 799 |
| **33** | Ceylon | Sri Lanka | Bark | ND | 22.7 | ND | ND | ND | 67.5 | ND | ND | ND | ND | ND | 224 | 69.9 |
| **34** | Ceylon | Sri Lanka | Bark | 4.17 | 6.66 | ND | ND | ND | 89.1 | ND | ND | ND | ND | ND | 420 | 67.3 |
| **35** | Ceylon | Sri Lanka | Bark | ND | 42.6 | ND | ND | ND | 44.4 | ND | ND | ND | ND | ND | 257 | 145 |
| **36** | Ceylon | Sri Lanka | Bark | 1.90 | 174 | ND | ND | ND | 71.2 | ND | ND | ND | ND | ND | 588 | 184 |
| **37** | Ceylon | NA | Bark | ND | 85.8 | ND | ND | ND | 83.5 | ND | ND | ND | ND | ND | 631 | 977 |
| **38** | NA | NA | Bark | ND | ND | ND | ND | ND | 466 | ND | ND | ND | ND | ND | 302 | ND |
| **39** | Ceylon | Sri Lanka | Bark | ND | 409 | ND | ND | ND | 259 | 3.61 | ND | 13.6 | ND | ND | 2019 | 178 |
| **40** | Ceylon | NA | Ground | ND | 28.2 | 14.6 | ND | 134 | 845 | 70.6 | 104 | ND | ND | 43.0 | 80.0 | ND |
| **41** | Ceylon | Sri Lanka | Ground | ND | 336 | ND | ND | ND | 52.4 | ND | ND | ND | ND | ND | 812 | 385 |
| **42** | Ceylon | Sri Lanka | Bark | ND | 80.3 | ND | ND | ND | 359 | 5.19 | ND | ND | ND | ND | 1927 | 33.5 |
| **43** | Ceylon | NA | Ground | ND | ND | ND | ND | ND | 148 | ND | ND | ND | ND | ND | 400 | ND |
| **44** | Ceylon | Madagascar | Ground | 4.67 | 167 | ND | ND | ND | 34.9 | ND | ND | ND | ND | ND | 181 | 22.4 |
| **45** | Ceylon | Sri Lanka | Ground | 6.03 | 10.8 | ND | 16.7 | ND | 232 | 6.15 | ND | 10.1 | ND | ND | 1968 | ND |
| **46** | Ceylon | India | Ground | ND | 123 | 8.36 | ND | 89.0 | 803 | 47.1 | 68.6 | ND | 93.9 | ND | 80.0 | 14.9 |
| **47** | Ceylon | NA | Bark | ND | 150 | ND | ND | ND | 109 | ND | ND | ND | ND | ND | 1331 | 1135 |
| **48** | Ceylon | Sri Lanka | Bark | ND | 33.4 | ND | ND | ND | 223 | 3.34 | ND | 26.5 | ND | ND | 1074 | ND |
| **49** | Ceylon | NA | Ground | 5.42 | 53.9 | ND | ND | ND | 4.22 | ND | ND | 4.42 | ND | ND | 44.6 | 16.2 |
| **50** | Ceylon | Sri Lanka | Ground | ND | ND | ND | 18.2 | 16.9 | 339 | 10.0 | 3.72 | ND | 97.0 | ND | 88.5 | ND |
| **51** | Ceylon | Sri Lanka | Ground | ND | 10.4 | ND | ND | ND | 268 | 6.76 | ND | 12.7 | ND | ND | 2020 | ND |
| **52** | Cassia | Indonesia | Bark | ND | ND | ND | ND | 40.2 | 1005 | 5.78 | 2.75 | ND | 172 | ND | 613 | 4.57 |

e)

| **Sample id** | **Label variety** | **Origin** | **Bark/ground** | **humulene** | **alloaromadendrene** | **γ-muurolene** | **α-curcumene** | **selinene** | **α-muurolene** | **β-bisabolene** | **calamenene** | **isolongifolene dehydro** | **α-calacorene** | **caryophyllene oxide** | **caryophyllenyl alcohol** | **gleenol** |
| --- | --- | --- | --- | --- | --- | --- | --- | --- | --- | --- | --- | --- | --- | --- | --- | --- |
| **1** | NA | NA | Ground | 7.47 | ND | 90.4 | ND | 48.2 | 288 | ND | 295 | 13.7 | 22.2 | ND | ND | 14.0 |
| **2** | NA | NA | Ground | ND | ND | 68.2 | ND | 26.9 | 11.0 | ND | 194 | 9.27 | 20.8 | ND | ND | 5.33 |
| **3** | NA | NA | Ground | 25.6 | ND | 97.0 | ND | ND | 429 | 3.74 | 307 | 14.4 | 30.7 | 7.38 | ND | 8.34 |
| **4** | NA | NA | Ground | 9.64 | 9.89 | 61.5 | ND | 24.6 | 419 | 6.48 | 325 | 25.2 | 60.0 | ND | 6.81 | 8.31 |
| **5** | NA | NA | Ground | 142 | 466.41 | 913 | ND | ND | 1359 | 77.4 | 820 | 295 | 392 | 4.63 | ND | 65.5 |
| **6** | NA | NA | Ground | 67.9 | ND | 130 | ND | 43.5 | 638 | 6.68 | 495 | ND | 79.9 | ND | ND | 12.6 |
| **7** | NA | NA | Ground | 8.98 | ND | 69.3 | ND | 32.2 | 265 | ND | 217 | 23.9 | 45.0 | ND | 5.94 | 11.5 |
| **8** | NA | NA | Ground | 97.0 | ND | 265 | ND | 13.8 | 1254 | 10.0 | 906 | 64.8 | 148 | ND | 17.6 | 22.9 |
| **9** | NA | NA | Ground | 40.7 | 3.09 | 119 | ND | 45.0 | 595 | 7.00 | 416 | 28.2 | 76.0 | ND | ND | 11.2 |
| **10** | NA | NA | Ground | 57.1 | ND | 865 | 398.44 | 153 | 1309 | 232 | 882 | 112 | 287 | ND | ND | 39.0 |
| **11** | Ceylon | Madagascar | Ground | 3.61 | ND | ND | 2.85 | ND | ND | ND | 6.02 | ND | ND | 5.19 | 35.7 | ND |
| **12** | Ceylon | Sri Lanka | Ground | 17.1 | ND | 4.72 | 8.07 | 3.80 | 14.4 | ND | 14.9 | ND | 2.02 | 6.11 | 33.2 | ND |
| **13** | NA | NON EU | Ground | 11.0 | 13.32 | 94.5 | ND | ND | 534 | ND | 309 | 21.0 | 64.5 | ND | 9.68 | 11.8 |
| **14** | Ceylon | Madagascar | Ground | 13.9 | ND | ND | 11.43 | ND | 4.19 | ND | 6.39 | ND | ND | 3.59 | 25.6 | ND |
| **15** | Burmanni | NA | Ground | 2.55 | ND | 18.0 | ND | ND | 60.9 | ND | 82.1 | ND | 21.9 | ND | ND | 9.39 |
| **16** | Aromaticum | NA | Ground | 50.8 | ND | 142 | ND | 62.1 | 496 | 4.73 | 430 | 26.9 | 63.1 | ND | ND | 14.6 |
| **17** | NA | Ceylan and Indonesia | Ground | 29.4 | ND | 252 | ND | ND | 669 | 58.7 | 507 | 43.1 | 69.0 | 6.67 | ND | 13.0 |
| **18** | Ceylon | NON EU | Ground | 13.9 | ND | ND | 9.51 | ND | ND | ND | 15.4 | ND | ND | 3.15 | 15.1 | ND |
| **19** | Ceylon | NON EU | Ground | ND | ND | 450 | ND | ND | 309 | 47.8 | 506 | ND | 197 | ND | 26.0 | 42.4 |
| **20** | Ceylon | NON EU | Ground | 20.0 | ND | ND | 16.96 | ND | 6.71 | ND | 14.3 | ND | ND | 5.09 | 19.7 | ND |
| **21** | Ceylon | NA | Ground | 2.22 | ND | 5.47 | ND | ND | 16.8 | ND | 23.6 | ND | 8.58 | ND | 4.06 | ND |
| **22** | Ceylon | NA | Ground | 3.05 | ND | ND | 2.79 | ND | ND | ND | 2.21 | ND | ND | ND | 5.08 | ND |
| **23** | Cassia | NA | Ground | ND | ND | 161 | ND | 78.9 | 472 | ND | 283 | 32.6 | 90.4 | ND | 24.7 | 18.1 |
| **24** | Cassia | Vietnam | Ground | 123 | ND | 626 | ND | ND | 1320 | 203 | 769 | 136 | 277 | ND | 24.3 | 37.5 |
| **25** | Ceylon | NA | Ground | 97.2 | ND | 5.54 | 46.98 | ND | 18.4 | 4.48 | 24.0 | ND | 2.81 | 22.7 | 13.4 | ND |
| **26** | NA | Vietnam | Ground | 2.06 | ND | 16.5 | 11.87 | ND | 48.4 | 5.67 | 75.0 | 7.81 | 27.5 | ND | 4.80 | 6.34 |
| **27** | Burmanni | ND | Ground | ND | ND | 165 | ND | 39.8 | 664 | 14.3 | 455 | 34.7 | 66.8 | ND | 11.2 | 16.8 |
| **28** | Burmanni | ND | Ground | 20.0 | ND | 76.4 | ND | 32.4 | 356 | 5.57 | 321 | 27.0 | 55.2 | ND | 4.48 | 9.38 |
| **29** | Ceylon | NA | Ground | 50.8 | ND | 9.35 | 15.62 | 8.62 | 25.0 | ND | 36.0 | ND | 2.94 | 8.29 | 22.0 | ND |
| **30** | Cassia | NA | Ground | 30.1 | ND | 85.5 | 56.81 | 36.0 | 311 | ND | 250 | 16.5 | 38.7 | ND | ND | 14.9 |
| **31** | NA | Sri Lanka | Ground | 39.3 | ND | 180 | 65.04 | 54.8 | 771 | 22.4 | 486 | 35.3 | 85.3 | ND | 11.8 | 16.4 |
| **32** | Ceylon | Sri Lanka | Bark | 134 | ND | 2.27 | 8.88 | ND | 7.47 | ND | 6.34 | ND | ND | 13.1 | 32.3 | ND |
| **33** | Ceylon | Sri Lanka | Bark | 35.1 | ND | ND | 5.47 | ND | 3.37 | ND | 6.28 | ND | ND | 4.69 | 9.91 | ND |
| **34** | Ceylon | Sri Lanka | Bark | 62.6 | ND | ND | 10.66 | ND | 4.77 | ND | 6.95 | ND | ND | 4.14 | 20.3 | ND |
| **35** | Ceylon | Sri Lanka | Bark | 41.1 | ND | ND | 3.96 | ND | 1.84 | ND | ND | ND | ND | 28.2 | 13.9 | ND |
| **36** | Ceylon | Sri Lanka | Bark | 92.0 | ND | ND | 4.27 | ND | 2.13 | ND | ND | ND | ND | 24.7 | 6.69 | ND |
| **37** | Ceylon | NA | Bark | 92.6 | ND | ND | 6.47 | ND | ND | ND | ND | ND | ND | 16.3 | 8.68 | ND |
| **38** | NA | NA | Bark | 43.7 | ND | ND | ND | ND | 8.72 | ND | 16.4 | ND | 5.36 | 152 | ND | ND |
| **39** | Ceylon | Sri Lanka | Bark | 686 | ND | ND | 9.46 | ND | ND | ND | 6.07 | ND | ND | 13.0 | 16.4 | ND |
| **40** | Ceylon | NA | Ground | 68.3 | ND | 499 | 242.25 | ND | 657 | 199 | 637 | ND | 232 | ND | 26.4 | 21.4 |
| **41** | Ceylon | Sri Lanka | Ground | 149 | ND | ND | 11.06 | ND | ND | ND | 5.00 | ND | ND | 14.5 | 7.40 | ND |
| **42** | Ceylon | Sri Lanka | Bark | 490 | 8.34 | 5.54 | 20.44 | ND | 10.4 | ND | 7.24 | ND | ND | 25.4 | 10.2 | ND |
| **43** | Ceylon | NA | Ground | 85.3 | ND | ND | 24.77 | ND | 9.54 | ND | ND | ND | 3.13 | 23.4 | ND | ND |
| **44** | Ceylon | Madagascar | Ground | 26.2 | ND | ND | 13.13 | ND | 4.71 | ND | 11.8 | ND | ND | 13.2 | 20.8 | ND |
| **45** | Ceylon | Sri Lanka | Ground | 562 | ND | 5.72 | 48.95 | 36.1 | ND | 5.12 | 29.5 | ND | ND | 52.9 | 37.8 | ND |
| **46** | Ceylon | India | Ground | 66.6 | ND | 382 | 257.15 | 97.9 | 800 | 390 | 528 | 103 | 41.5 | ND | ND | 13.9 |
| **47** | Ceylon | NA | Bark | 169 | ND | ND | 6.57 | ND | 3.72 | ND | 3.50 | ND | ND | 15.8 | 7.83 | ND |
| **48** | Ceylon | Sri Lanka | Bark | 296 | ND | ND | 10.92 | ND | 4.94 | ND | 7.26 | ND | ND | 15.4 | 17.6 | ND |
| **49** | Ceylon | NA | Ground | 5.79 | ND | ND | 3.81 | ND | ND | ND | 4.20 | ND | ND | 6.55 | 12.3 | ND |
| **50** | Ceylon | Sri Lanka | Ground | 17.0 | ND | 87.6 | ND | ND | 401 | ND | 302 | 21.5 | 56.2 | ND | 4.88 | 6.55 |
| **51** | Ceylon | Sri Lanka | Ground | 601 | ND | 5.77 | 47.01 | 38.1 | ND | ND | 26.7 | ND | ND | 49.4 | 34.1 | ND |
| **52** | Cassia | Indonesia | Bark | 82.6 | ND | 42.5 | 30.73 | ND | 158 | 4.90 | 125 | ND | 15.6 | 8.53 | ND | 3.74 |

f)

| **Sample id** | **Label variety** | **Origin** | **Bark/ground** | **tetradecanal** | **cubenol** | **δ-cadinol** | **ar-tumerone** | **cadalene** | **tetradecanoic acid, methyl ester** | **palmitic acid, methyl ester** | **linoleic acid, methyl ester** | **oleic acid, methyl ester** | **stearic acid, methyl ester** |
| --- | --- | --- | --- | --- | --- | --- | --- | --- | --- | --- | --- | --- | --- |
| **1** | NA | NA | Ground | ND | 92.0 | 65.0 | ND | 73.0 | ND | 3.64 | ND | ND | ND |
| **2** | NA | NA | Ground | ND | 46.4 | 59.9 | ND | 40.8 | 1.71 | 26.0 | 5.07 | 60.3 | ND |
| **3** | NA | NA | Ground | ND | ND | 97.5 | ND | 85.7 | ND | ND | ND | ND | ND |
| **4** | NA | NA | Ground | 14.3 | 86.9 | 86.8 | ND | 35.3 | ND | 10.5 | ND | 3.79 | ND |
| **5** | NA | NA | Ground | ND | 297 | 316 | ND | 112 | ND | ND | ND | ND | ND |
| **6** | NA | NA | Ground | ND | 71.9 | 58.5 | ND | 27.5 | ND | 25.8 | 2.07 | 6.08 | ND |
| **7** | NA | NA | Ground | ND | 73.0 | 5.18 | ND | 38.1 | ND | 10.7 | 3.29 | 8.70 | ND |
| **8** | NA | NA | Ground | ND | 125 | 189 | ND | 125 | ND | 17.4 | ND | 3.92 | ND |
| **9** | NA | NA | Ground | ND | 68.5 | 73.8 | ND | 33.2 | ND | 36.1 | 3.56 | 9.00 | ND |
| **10** | NA | NA | Ground | ND | 131 | 354 | ND | 224 | ND | 42.4 | 1.82 | 9.82 | ND |
| **11** | Ceylon | Madagascar | Ground | 37.2 | ND | ND | ND | ND | 22.6 | 149 | ND | 12.0 | ND |
| **12** | Ceylon | Sri Lanka | Ground | 19.6 | ND | ND | ND | 5.21 | ND | 12.3 | ND | 47.7 | ND |
| **13** | NA | NON EU | Ground | 57.6 | 87.5 | 195 | ND | 67.7 | 7.66 | 49.1 | ND | 15.0 | ND |
| **14** | Ceylon | Madagascar | Ground | 46.6 | ND | ND | ND | ND | ND | 15.0 | ND | ND | ND |
| **15** | Burmanni | NA | Ground | ND | 56.9 | 71.4 | ND | 32.5 | 3.60 | 47.0 | 2.31 | 10.6 | ND |
| **16** | Aromaticum | NA | Ground | ND | 97.0 | 71.5 | ND | 31.4 | ND | 71.9 | 6.91 | 17.7 | ND |
| **17** | NA | Ceylan and Indonesia | Ground | ND | 72.3 | 15.0 | ND | 105 | ND | 29.9 | ND | 32.2 | ND |
| **18** | Ceylon | NON EU | Ground | 44.9 | ND | ND | ND | ND | 51.6 | 487 | 17.0 | 69.4 | 8.56 |
| **19** | Ceylon | NON EU | Ground | ND | 118 | 389 | ND | 96.3 | ND | 3.03 | ND | ND | ND |
| **20** | Ceylon | NON EU | Ground | 35.5 | ND | ND | ND | ND | 63.8 | 653 | 25.2 | 108 | 14.6 |
| **21** | Ceylon | NA | Ground | 18.8 | ND | 31.6 | 31.0 | 4.95 | ND | 27.5 | ND | 62.2 | ND |
| **22** | Ceylon | NA | Ground | 23.9 | ND | ND | 7.89 | ND | 2.83 | 25.4 | ND | 2.68 | ND |
| **23** | Cassia | NA | Ground | 222 | ND | 175 | ND | 65.3 | 10.3 | 20.0 | ND | 3.41 | 2.15 |
| **24** | Cassia | Vietnam | Ground | ND | 147 | 339 | ND | 95.8 | ND | 22.2 | 1.65 | 4.91 | ND |
| **25** | Ceylon | NA | Ground | 45.0 | ND | ND | ND | ND | ND | 4.76 | ND | ND | ND |
| **26** | NA | Vietnam | Ground | 30.7 | 41.6 | 78.4 | 27.8 | 13.7 | ND | 36.8 | 1.85 | 10.5 | ND |
| **27** | Burmanni | ND | Ground | ND | 55.9 | 137 | ND | 77.4 | ND | ND | ND | ND | ND |
| **28** | Burmanni | ND | Ground | ND | 72.5 | 72.7 | ND | 30.8 | ND | 5.92 | ND | ND | ND |
| **29** | Ceylon | NA | Ground | 59.4 | ND | ND | ND | 10.5 | 39.1 | 126 | 3.90 | 28.1 | 2.79 |
| **30** | Cassia | NA | Ground | ND | 112 | 106 | ND | 51.6 | ND | 125 | 11.3 | 29.8 | 3.08 |
| **31** | NA | Sri Lanka | Ground | ND | 102 | 184 | ND | 110 | ND | 15.3 | ND | 2.73 | ND |
| **32** | Ceylon | Sri Lanka | Bark | 49.3 | ND | ND | ND | ND | ND | ND | ND | ND | ND |
| **33** | Ceylon | Sri Lanka | Bark | 45.2 | ND | ND | ND | ND | ND | 5.93 | ND | ND | ND |
| **34** | Ceylon | Sri Lanka | Bark | 55.4 | ND | ND | ND | ND | ND | 5.99 | ND | ND | ND |
| **35** | Ceylon | Sri Lanka | Bark | 63.8 | ND | ND | ND | ND | ND | 6.23 | ND | ND | ND |
| **36** | Ceylon | Sri Lanka | Bark | 48.0 | ND | ND | ND | ND | ND | 5.72 | ND | ND | ND |
| **37** | Ceylon | NA | Bark | 39.0 | ND | ND | ND | ND | ND | 6.91 | ND | ND | ND |
| **38** | NA | NA | Bark | ND | ND | ND | ND | ND | ND | 4.79 | ND | ND | ND |
| **39** | Ceylon | Sri Lanka | Bark | 22.0 | ND | ND | ND | ND | ND | ND | ND | ND | ND |
| **40** | Ceylon | NA | Ground | ND | 119 | 292 | ND | 58.4 | ND | 9.12 | 1.13 | 2.24 | ND |
| **41** | Ceylon | Sri Lanka | Ground | 25.2 | ND | ND | ND | ND | ND | 91.7 | 9.91 | 14.5 | ND |
| **42** | Ceylon | Sri Lanka | Bark | 60.5 | ND | ND | ND | ND | ND | 36.4 | ND | ND | ND |
| **43** | Ceylon | NA | Ground | 113 | ND | ND | ND | ND | 203 | 413 | 24.5 | 85.2 | 14.6 |
| **44** | Ceylon | Madagascar | Ground | 24.0 | ND | ND | 9.47 | ND | 24.9 | 247 | 32.1 | 53.0 | 3.57 |
| **45** | Ceylon | Sri Lanka | Ground | 59.2 | ND | ND | ND | ND | 89.8 | 665 | 73.9 | 158 | 18.5 |
| **46** | Ceylon | India | Ground | ND | 76.7 | 144 | ND | 37.5 | ND | 22.0 | 1.93 | 4.40 | ND |
| **47** | Ceylon | NA | Bark | 42.6 | ND | ND | ND | ND | ND | ND | ND | ND | ND |
| **48** | Ceylon | Sri Lanka | Bark | 46.8 | 1.63 | ND | ND | ND | ND | 22.7 | 3.09 | 2.87 | ND |
| **49** | Ceylon | NA | Ground | 13.7 | ND | ND | ND | ND | 14.3 | 180 | 20.1 | 44.6 | 4.14 |
| **50** | Ceylon | Sri Lanka | Ground | ND | 81.3 | 137 | ND | 47.9 | ND | 35.4 | ND | 6.35 | ND |
| **51** | Ceylon | Sri Lanka | Ground | 59.6 | ND | ND | ND | ND | 87.9 | 635 | 65.7 | 146 | 16.4 |
| **52** | Cassia | Indonesia | Bark | ND | 33.7 | 24.5 | ND | 3.93 | ND | 19.9 | 3.82 | 6.34 | ND |
